# “The Heisenberg Method”: Geometry, Algebra, and Probability in Quantum Theory

**DOI:** 10.3390/e20090656

**Published:** 2018-08-30

**Authors:** Arkady Plotnitsky

**Affiliations:** Literature, Theory, Cultural Studies Program, Purdue University, West Lafayette, IN 47907, USA; plotnits@purdue.edu

**Keywords:** algebra, causality, geometry, probability, quantum information theory, realism, reality

## Abstract

The article reconsiders quantum theory in terms of the following principle, which can be symbolically represented as *QUANTUMNESS* → *PROBABILITY* → *ALGEBRA* and will be referred to as the QPA principle. The principle states that the quantumness of physical phenomena, that is, the specific character of physical phenomena known as quantum, implies that our predictions concerning them are irreducibly probabilistic, even in dealing with quantum phenomena resulting from the elementary individual quantum behavior (such as that of elementary particles), which in turn implies that our theories concerning these phenomena are fundamentally *algebraic*, in contrast to more geometrical classical or relativistic theories, although these theories, too, have an algebraic component to them. It follows that one needs to find an algebraic scheme able make these predictions in a given quantum regime. Heisenberg was first to accomplish this in the case of quantum mechanics, as matrix mechanics, whose matrix character testified to his algebraic method, as Einstein characterized it. The article explores the implications of the Heisenberg method and of the QPA principle for quantum theory, and for the relationships between mathematics and physics there, from a nonrealist or, in terms of this article, “reality-without-realism” or RWR perspective, defining the RWR principle, thus joined to the QPA principle.

Perhaps the success of the Heisenberg method points to a purely algebraic method of description of nature, that is, to the elimination of continuous functions from physics. Then, however, we must give up, in principle, the space–time continuum.

—Albert Einstein, “Physics and Reality” (1936)

## 1. Introduction

This article reconsiders quantum theory, from quantum mechanics to quantum field theory to quantum information theory, primarily focusing on quantum mechanics, in terms of the following principle, which can be symbolically represented as:

*QUANTUMNESS* → *PROBABILITY* → *ALGEBRA*

and will be referred to as the QPA principle. This principle states, first, defining the experimental nature of my first implication, *QUANTUMNESS* → *PROBABILITY*, that the quantumness of physical phenomena, that is, the specific character of physical phenomena known as quantum, implies that our predictions concerning them are irreducibly probabilistic or statistical, even in dealing with quantum phenomena resulting from the elementary individual quantum behavior (such as that of elementary particles). This, in turn implies, defining the theoretical character on my second implication, *PROBABILITY* → *ALGEBRA*, that our theories concerning these phenomena, quantum theories, are fundamentally algebraic, in contrast to more geometrical classical or relativistic theories. Although this distinction is not unconditional, because quantum theories do have geometrical aspects, while, conversely, geometrical theories have algebraic aspects, it is, I shall argue, irreducible; and understanding this distinction, along with the shared aspects of both types of theories, helps us to shed new light on the relationships between algebra and geometry in physics. 

Physically, quantum phenomena are defined by the fact that, in considering them, Planck’s constant, *h*, must be taken into account, which allows one to use the classical theory in describing them, but not in predicting them. By “quantum physics” I shall refer to the overall assembly of the available quantum phenomena and theoretical accounts of these phenomena. The terms “classical physics” will be used along parallel lines for classical phenomena, which need not depend on *h* (or on *c*, the role of which defines relativistic phenomena). While, however, the role of *h* is irreducible in quantum phenomena, their specificity as quantum is defined by a broader set of physical features, such as the uncertainty relations (which do contain *h*), complementarity, and quantum correlations, some of which are not linked to *h*, at least not expressly. On the other hand, some of these features, although not all of them, are also exhibited by classical phenomena or found in mathematical models different from those of the standard quantum mechanics or quantum field theory. The ultimate distinction between quantum and classical phenomena is the subject of ongoing investigations and debates, on which subject I shall further comment below. From present perspective, *h* may not pertain to quantum objects or behavior but only to our theories, and enters these theories via the interactions between quantum objects and measuring instruments.

Quantum phenomena also obey the principle of discreteness or the QD principle, which, necessarily coupled to the individuality of quantum phenomena, may indeed represent the “essence” or “quantumness” of quantum theory, according to Bohr [1]. In other words, quantum phenomena are individual and discrete in relation to each other, which, as emphasized by N. Bohr, is not the same as the atomic, Democritean, discreteness of elementary quantum objects themselves, which was initially (following Planck’s discovery of quantum physics in 1900) seen as defining quantum physics as quantum [2]. By “elementary” I refer to those quantum objects, also known as “elementary particles,” that cannot be considered as composite. Either character, elementary or composite, could be ascertained on the basis of effects such objects have on measuring instruments, keeping in mind that some particles considered elementary can reveal themselves to be composite, as it happened in the case of hadrons that were found to be composed of quarks and gluons. 

It follows that the difference between objects and phenomena is irreducible in quantum theory, as opposed to classical theory, specifically classical mechanics, which deals with individual classical objects or simple classical systems. Rigorously speaking, as Kant already realized, this difference exists there as well, but it can be disregarded insofar as we can, at least ideally and in principle, consider such objects by neglecting the interference of observation. This is not possible in quantum physics, at least not explicitly. It is under debate whether it is possible indirectly, inferentially, to establish the independent nature and behavior of quantum objects, similarly to the way in which we can treat, by means of classical mechanics, the nature and behavior of the elemental constituents of the systems considered in classical statistical physics, even though we do not directly observe this behavior. (This approach helped the nineteenth-century physics to confirm the existence of atoms.) The interpretation of quantum phenomena adopted in this article, following Bohr and Heisenberg (in his early work, discussed below [3]), precludes this possibility of attributing any independent properties to quantum objects, although it does not preclude alternative interpretations of quantum phenomena. The QPA principle would still hold for most of these interpretations, possibly, in contrast to the present view, under the assumption of a continuously connected underlying reality. There is, thus, another implication: *QUANTUMNESS* → *DISCRETENESS*. I shall, however, subsume the discreteness of quantum phenomena under quantumness.

I shall discuss the concepts of “geometry,” “algebra,” and “probability,” and the relationships between them in more detail below. Briefly, I understand geometry as the mathematical formalization of spatiality in terms of measurement (while topology as referring to the structure of spatiality apart from measurement), algebra as the mathematical formalization of the relationships between symbols, arithmetic as dealing with numbers, and probability as the mathematical formalization the likelihood of events and expectations concerning them (the corresponding mathematical fields are geometry, topology, algebra, number theory, and probability theory). Geometrical and topological objects always have algebraic components, while algebraic objects need not have a geometrical component.

Now, there is next to nothing geometrical about probability or probability theory. The origin of probability theory coincides with the rise of algebra, in the works of Cardano, Fermat, Descartes, and Pascal. Some form of algebra was necessary for probability theory, as Hacking persuasively argued in explaining why the theory emerged in the seventeenth century rather than earlier [4]. Analytic geometry and calculus were introduced around the same time by, the first by Fermat and Descartes, and the second by Newton and Leibniz (although Fermat was, again, an important precursor, especially as concerns the algebraic aspects of calculus), and these fields, too, were the product of the algebraization of mathematics, a defining feature of the mathematics and physics of modernity, even though geometry continued to dominate both until the nineteenth century.

It is true that probability theory, uses spatialized mathematical concepts, such as that of “probability space,” introduced by Kolmogorov as part of his axiomatization of probability theory, just as quantum mechanics uses the concept of “Hilbert space.” Kolmogorov’s concept follows the concepts of “space” developed in functional analysis and measure theory (which Kolmogorov used to axiomatize probability theory [5]), that of Hilbert space, among them. As I shall argue, however, these concepts are more algebraic than geometrical: They have algebraic structures that geometrical objects possess but are not aimed at representing the physical space, the original and still continuing task of geometry, even though it has, as a mathematical field, developed far beyond its concerns with nature or its relations to physics.

Quantum mechanics reshaped the relationships between the algebra of probability and the algebra of theoretical physics, as against previous uses of probability, for example, in classical statistical physics. There the relationships between them is underlain by a geometrical picture of the behavior of the individual constituents of the systems considered, assumed to follow the laws of classical mechanics. By contrast, as became apparent beginning with Planck’s discovery of quantum phenomena, even elementary individual quantum objects and the events they give rise to had to be treated probabilistically. One needed, accordingly, to find a new theory to make correct probabilistic or statistical predictions concerning them, a task that quantum theory pursued from its inception, with mixed results. Heisenberg was able to accomplish this task with quantum mechanics as matrix mechanics, which avoided the deficiencies of “the old quantum theory,” as it became called after the introduction of quantum mechanics, and which only predicted the probabilities of what was observed in measuring instruments, as quantum phenomena, without describing the behavior of quantum objects [3]. Heisenberg’s use of his matrix variables as operators in linear vector spaces (essentially, infinite-dimensional Hilbert spaces over **C**) defined the algebraic nature of “the Heisenberg method,” as Einstein characterized it [6]. This was in contrast to Schrödinger’s more geometrical method in his wave mechanics, accompanied by a geometrical conception of quantum-level reality in terms of a continuous vibrational process, a conception never worked out by Schrödinger to accord with the experimentally established discrete features of quantum phenomena. Indeed, the physical and mathematical demands of accounting for these features led Schrödinger to a mathematically equivalent scheme [7]. I shall explain below why Schrödinger’s equation may be seen in probabilistically predictive terms, without (geometrically) representing either the propagation of wave or the motion of particles in space and time. 

Bohr, in his initial assessment of Heisenberg’s discovery in 1925, before Schrödinger’s wave version, but after Born and Jordan’s paper, which gave Heisenberg’s initial scheme its proper matrix form [8], explained Heisenberg’s approach as follows [9]:

In contrast to ordinary mechanics, *the new quantum mechanics does not deal with a space–time description of the motion of atomic particles*. It operates with manifolds of quantities which replace the harmonic oscillating components of the motion and symbolize the possibilities of transitions between stationary states in conformity with the correspondence principle [which requires that quantum and classical predictions coincide in the classical limit]. These quantities satisfy certain relations which take the place of the mechanical equations of motion and the quantization rules [of the old quantum theory]. (emphasis added)

Thus, in effect combining the QPA principle with another principle, the *reality-without-realism*, RWR, principle (defined by the fact that “the new quantum mechanics does not deal with a space–time description of the motion of atomic particles” and explained in detail below), Heisenberg’s quantum mechanics was a radical departure from the preceding history of modern (mathematical-experimental) physics, from Galileo’s mechanics to Einstein’s relativity and even to the previous quantum theory. All these theories were based on such descriptions or representations, either phenomenally visualizable, usually on geometrical *lines* (referring to the continuous motion of the objects considered), at least in dealing with elementary individual processes, as in classical mechanics, or beyond visualization but given a conceptual or mathematical representation, as in relativity when dealing with photons and velocities close to *c*. Classical statistical physics still relied and even depended on the representational treatment of the behavior of the elemental constituents of the multiplicities considered, constituents that were viewed as behaving in accordance with the laws of classical mechanics. The description of individual quantum objects and the corresponding mathematical representation became partial in the so-called old quantum theory, such as Bohr’s atomic theory, introduced in 1913 and developed by him and others over the following decade [10,11]. The old (semiclassical) quantum theory only provided such a representation, in terms of orbits, for stationary states of electrons in atoms, but not for the discrete transitions, “quantum jumps,” between stationary states. The relativistic law of addition of velocities (defined by the Lorentz transformation) in special relativity, $s=\frac{v+u}{1+{\left(vu/c\right)}^{2}}$, for collinear motion (*c* is the speed of light in a vacuum), runs contrary to any intuitive (geometrical) representation of motion that we can have. This concept of motion is, thus, no longer a mathematical refinement of a daily concept of motion in the way the classical concept of motion is. Relativity was the first physical theory that defeated our ability to form a phenomenal conception of an elementary physical process, and it was a radical change in the history of physics. The reason that this aspect of relativity worried Einstein less than quantum mechanics was that relativity still offered a conceptual, as well as mathematical, representation of the behavior of individual systems, which could, moreover, be handled deterministically rather than probabilistically. Besides, there was plenty of geometry in the theory, all the more so in general relativity, the geometry grounded in the spacetime continuum, which Einstein wanted to preserve as part of physical reality at all levels and which was threatened by the Heisenberg method [6]. Ultimately, photons are quantum objects and are treated by quantum electrodynamics, which, in the present view, no longer represents the behavior of quantum objects in the corresponding (high-energy) regimes any more than quantum mechanics.

Heisenberg abandoned a geometrical representation of stationary states retained in Bohr’s 1913 theory and, thus, any description or representation, even a mathematical one, of *the behavior of quantum objects*, in accordance with the RWR principle. Stationary states were only represented algebraically by energy-level values, considered apart from representing the behavior of electrons themselves to which these energy levels were assigned. One was no longer thinking, as in classical physics or relativity, in terms of predicting, even in considering elementary individual processes, the spacetime behavior of the objects considered due to (continuous) changes in their states, assumed to be definable independently of the interactions between objects and measuring instruments, and representable by the corresponding formalism. Instead, following Bohr’s thinking concerning quantum jumps in his 1913 atomic theory, one was thinking in terms of discontinuous transitions between physical states of quantum objects and the probabilities or statistics of such transitions, as only probabilistic predictions were possible experimentally [12]. These states are, moreover, only manifested as effects of the interactions between quantum objects and measuring instruments. While not part of Bohr’s 1913 theory, this understanding became central to Bohr’s thinking following Heisenberg’s discovery and came to define Bohr’s interpretation of quantum phenomena and quantum mechanics. (Although quantum phenomena and quantum mechanics are commonly interpreted jointly, as they were by Bohr or are here, quantum phenomena could be given an interpretation independent of a theory accounting for them.)

Heisenberg’s theory may be seen in terms of transition from geometry to algebra in fundamental physics, which was acutely sensed by Einstein, who was hardly welcoming this transition [6]:

[P]erhaps the success of the Heisenberg method points to a purely algebraic method of description of nature, that is, to the elimination of continuous functions from physics. Then, however, we must give up, in principle, the space–time continuum [at the ultimate level of reality]. It is not unimaginable that human ingenuity will some day find methods which will make it possible to proceed along such a path. At present however, such a program looks like an attempt to breathe in empty space.

Einstein was equally unhappy with the recourse to probability in dealing with elementary individual processes, and he thought it would be avoided by a kind of fundamental theory he envisioned, a continuous geometrical field theory of the type general relativity was. Earlier, he referred to Heisenberg’s scheme as a magical trick, “Jacob’s pillow,” of Göttingen: it was not “the real thing” and “[did] not really bring us any closer to the secret of the ‘old one,’” who, Einstein added in his famous pronouncement, “at any rate is … not playing at dice” [13]. Ultimately, Einstein was more concerned with the absence of realism at the fundamental level. As, however, he must have realized, randomness and the recourse to probability are automatic in this absence. 

I shall also argue here, as a bridge to considering quantum information theory, that, while not, technically, quantum-informational, Heisenberg’s thinking could be viewed as quantum-informational *in spirit*, and conversely, quantum information theory as Heisenbergian in spirit, and thus both as algebraic in spirit [14,15]. The reason for this view is that the quantum-mechanical situation, as Heisenberg conceived of it, was, in retrospect, defined by:

(a) certain *already obtained* information, concerning the energy of an electron, derived from spectral lines (due to the emission of radiation by the electron), *observed* in measuring instruments; and
(b) certain possible future information, concerning the energy of this electron, *to be obtainable* from spectral lines *to be observed* in measuring instruments and predictable (on experimental grounds) in probabilistic or statistical terms by the mathematical formalism of one or another quantum theory.

Heisenberg’s aim was to develop such a formalism without assuming that this formalism needed to represent a spatiotemporal process connecting these two sets of information or how each set comes about. Heisenberg’s quantum mechanics was about quantum information, albeit not only about it. It was equally about the nature of quantum objects, even though and because this nature was beyond human knowledge and even thought. But then, this is also true about much foundational thinking in quantum information theory, which aims to understand the ultimate nature of reality through the nature of quantum information.

The remainder of this article proceeds as follows: the next section outlines my main concepts. Section 3 addresses algebra and geometry in fundamental physics. Section 4 revisits Heisenberg’s discovery of quantum mechanics and Bohr’s interpretation of it. Section 5 considers some recent work in quantum information theory. 

## 2. Fundamentals of the QPA/RWR Approach to Quantum Theory

The currently standard version of quantum theory, the only one to be considered in this article, is comprised of three theories, all discovered in quick succession between 1925 and 1928. The first is quantum mechanics for continuous variables in infinite-dimensional Hilbert spaces (QM), the second is quantum theory for discrete variables in finite-dimensional Hilbert spaces (QTFD), and the third is quantum field theory in Hilbert spaces that are tensor products of finite and infinite dimensional Hilbert spaces (QFT), initially introduced in the form of quantum electrodynamics (QED). All these theories are algebraic and probabilistic or statistical and are governed by the QPA principle. QFT, which handles high-energy physics, is comprised of several theories, constituting the standard model of particle physics: quantum electrodynamics (QED), the theory of weak forces, and the theory of strong forces, quantum chromodynamics (QCD). While the first two are unified or (as some prefer to see it) “merged” in the electroweak theory, the unification of all three, known as “grand unification,” has not been achieved. More troubling is that QFT and general relativity are inconsistent with each other. This inconsistency is one of the greatest outstanding problems of fundamental physics, which motivated string and M-brane theories, and alternative approaches, including in quantum information theory (e.g., [16]).

The interpretation of quantum phenomena and quantum theory adopted here is defined by a nonrealist or “reality-without-realism” (RWR) view of quantum theory in any of its versions, a view that follows “the Copenhagen spirit of quantum theory” [*Kopenhagener Geist der Quantenheorie*], as Heisenberg called it [17]. This characterization, abbreviated here to “the spirit of Copenhagen,” is preferable to the more common “Copenhagen interpretation,” because there is no single such interpretation, even in the case of Bohr, who changed his views a few times [18] (here I shall be primarily concerned with the ultimate version of his interpretation). This is an important point. First, there is much confusion concerning this fact by both critics and advocates of Bohr and the spirit of Copenhagen. Secondly, at stake *are interpretations*, those (again, several) in the spirit of Copenhagen amidst still others, and not the ultimate truth of nature, which we do not know and may never know or even imagine and concerning which this article makes no definitive claims. In most of this article, I will be concerned with QM. I will give some attention to QTFD in the context of quantum information theory, which has been primarily concerned with it. QFT will only be mentioned in passing, although the QPA principle and the RWR principle apply there, and historically, QFT, beginning with QED, has been used to support the spirit of Copenhagen all along. I shall now outline the key concepts grounding my argument, in part in order to avoid misunderstandings concerning them, because these concepts or, more accurately, concepts designated by these terms can be defined otherwise. 

It is fitting to begin by addressing the concept of concept, first, because, it is rarely adequately considered in physical or philosophical literature, and secondly and more importantly, because the role of concepts is not sufficiently appreciated in the philosophy of physics, especially the analytic philosophy of physics. If, as Wilczek, a leading elementary particle theorist and a Nobel Prize laureate, argues, “the primary goal of fundamental physics is to discover profound concepts that illuminate our understanding of nature,” then creative thinking in fundamental physics is defined by concepts and is advanced by the discovery or invention of new concepts [19]. But what is a physical concept, and what is a concept in the first place? Wilczek does not explain it, taking it for granted or assuming some general sense of it presumably shared by his readers. One might safely assume, given the specific concepts that Wilczek invokes, such as that of “elementary particle” associated with that of “symmetry group,” that the concepts in question have mathematical components, the presence of which has defined the concepts of all modern, post-Galilean, theoretical physics. I shall also understand a physical theory as an organized assemblage of concepts in the sense about to be defined, an assemblage that relate certain physical objects or phenomena, usually in terms of propositions that are considered to be true, at least with a sufficient practical, even if not fully definitive, justification.

It is the latter aspect that tends to dominate the concepts of theory used in the analytic philosophy of physics. This aspect is of course indispensable: no physical theory, or philosophical argument concerning theoretical physics, can bypass it. I would, nevertheless, argue, following Borel’s 1907 critique of the logically based understanding of mathematics, which is, in my view, applicable to the logically based understanding of theoretical physics as well [20]. For Borel, a truly fertile invention in mathematics and theoretical physics alike consists of the discovery of new concepts that enable a new point of view from which to interpret the facts, followed by a search for the necessary proofs by plausible reasoning, and only then, necessarily, bringing logic in. According to Gray, “Borel’s criticisms point quite clearly toward a problem that has not gone away in philosophers’ treatment of mathematics: a tendency to reduce it to some essence than not only deprives it of purpose but is false to mathematical practice. The logical enterprise, even if it had succeeded, would only have been an account of part of mathematics, its deductive skeleton” [21]. This, I would contend, is true about much of the analytic philosophy of physics, again, indispensable as the propositional and logical aspects of theoretical physics are, in physics also as concerns correspondence with the available experimental evidence.

Bohr clearly understood the significance of these aspects of a physical theory and specifically QM, and used them in addressing Einstein’s criticism: “In my opinion, there could be no other way to deem a logically consistent mathematical formalism as inadequate than by demonstrating the departure of its consequences from experience or by proving that its predictions did not exhaust the possibilities of observation, and Einstein’s argumentation could be directed to neither of these ends” [22]. Bohr also understood, however, now in agreement with Einstein, that the invention of concepts play a decisive creative role in theoretical physics. Einstein saw “conceptual construction” [*begrrifliche Konstruction*] as essential to and irreducible in physics [23]. He also saw the practice of theoretical physics as that of the invention of new concepts through which one can approach reality, sometimes even to the point of overriding the experimental evidence [24]. Riemann, a major inspiration for Einstein’s general relativity, including as concerns conceptual construction, observed already in 1854: “From Euclid to Lagrange this darkness [in our understanding of the nature of geometry] has been dispelled neither by the mathematicians nor the philosophers who have concerned themselves with it. The reason [*Grund*] for this is undoubtedly because the general concept of multiply extended magnitudes, which includes spatial magnitudes, remains completely unexplored. I have therefore first set myself *the task of constructing the concept* of a multiply extended magnitude from general notions of magnitude” [25]. This led Riemann to his concept of manifold [*Maningfaltigkeit*], central to modern geometry and topology [26]. This article, too, while recognizing the indispensability of logical and propositional structures of physical theories, gives concepts and the invention of concepts the defining role the creative practice of theoretical physics.

I shall adopt the following understanding of concepts, in part following Deleuze and Guattari, whose thinking was inspired by Riemann and especially his concept of manifold [26,27]. In this definition, a concept is not merely a generalization from particulars (which is commonly assumed to define concepts) or a general or abstract idea, although a concept may contain such ideas, specifically abstract mathematical ideas in physics or in mathematics itself, where these ideas may become concepts in the present sense. A concept is a multicomponent entity, defined by the *organization* of its components, and some of these components may be concepts in turn. The definition may be very basic, but it reflects an essential character of concepts in any domain, from daily life to the stratosphere of mathematics and science. What is crucial is how this basic architecture is specifically instantiated in a given concept, which is defined by both the nature of the components and their organization, by how they relate to each other in the structure of the concept.

Consider as, an example, the concept of motion, first, as it is used in daily life: it will involve various components, such as a change of place, speed, acceleration, moving bodies, etc., which belong to our phenomenal intuition and are not defined rigorously, especially mathematically, but are still parts of the concept defined by the organization of these components. Now, one can, as both Bohr and Heisenberg did, see classical mechanics as a physical and mathematical refinement of these daily concepts by means of such mathematically defined concepts as coordinates, momentum, angular momentum, energy, and so forth, and thus also that of motion. While the concepts of classical physics are derived from the concepts of daily life, they are both mathematical and subject to an experimental verification. A concept could also be borrowed from a preceding physical theory and modified (or left intact). Every concept and every theory, no matter how innovative, has a history and depends on it. In quantum theory (QM, QFT or QTFD), in RWR-type interpretations, classical concepts are no longer applicable to quantum objects and their behavior, while they still have a limited applicability at the level of quantum phenomena, defined by effects of the interaction between quantum objects and measuring instruments. The concepts of quantum theory still have their history in classical physics, both physically (when applied to measuring instruments) and mathematically, for example, by adopting the concept of the Hamiltonian, while changing the variables from those of functions of real variables of classical mechanics to operator variables (in Hilbert spaces over **C**) in QM or QFT. The standard conservations laws (those of momentum, energy, and angular momentum), too, are preserved, although new conservation laws are added, such as the conservation of probability current in QM and QFT, or the conservation of baryon or lepton number in QFT. According to Heisenberg [28]:

The concepts of velocity, energy, etc., have been developed from simple experiments with common objects, in which the mechanical behavior of macroscopic bodies can be described by use of such words. The same concepts have then been carried over to the electron, since in certain fundamental experiments electrons show a mechanical behavior like that of the objects of common experience [or classical mechanics]. Since it is known, however, that this similarity exists only in a certain limited region of phenomena, the applicability of the corpuscular theory must be limited in the corresponding way…

As a matter of fact, it is experimentally certain only that light [too] sometimes behaves as if it possessed some of the attributes of a particle [as reflected in the uncertainty relations], but there is no experiment which proves that it possesses all the properties of a particle; similar statements hold for matter [e.g., electrons] and wave motion. The solution of the difficulty is that the two mental pictures [derived from classical physics] which experiments lead us to form—the one of particles, the other of waves—are both incomplete and have only the validity of analogies which are accurate only in limited cases. It is a trite saying that “analogies cannot be pushed too far,” but they may be justifiably used to describe things for which our language has no words. Light and matter are both single entities, and the apparent duality arises in the limitation of our language.

In the RWR-type view, quantum objects and behavior are beyond any representation, including mathematical one (which need not depend on language or physical concepts) or, in the view adopted here, are beyond conception. Either view transforms the wave-particle duality into the viewpoint defined by the concept complementarity, moreover, in the way in which, contrary to a common view of complementarity, there is no wave-particle complementarity. As Bohr noted, in referring to complementarity, a word that does not appear to have been used as *a noun* before Bohr (as opposed to the adjective “complementary”) and was introduced by Bohr to designate a new concept: “In the last resort an artificial word like ‘complementarity’ which does not belong to our daily concepts serves only briefly to remind us of the epistemological situation [found in quantum physics], which at least in physics is of an entirely novel character” [29]. Both the epistemological situation in question, essentially that of the RWR-type, and the architecture of the concept of complementarity, which does a great more than merely serving as such a reminder, will be discussed below. My main point at the moment is that complementarity is a new physical concept with several interrelated components. As most innovative concepts, complementarity, when introduced, was not defined by generalization from available entities: it was something entirely new, although it, too, had its history in physics and beyond [30,31]. It then functioned, in part, by generalizing multiple specific entities, such as specific complementary configurations, say, those of the position or the momentum measurement, always mutually exclusive at any given moment of time in the case of quantum phenomena. All physical concepts, including those of classical physics (which are closer to our daily concepts), are *physical* concepts, with mathematical components, ultimately divorced from their daily meaning and should be treated as such, which is not always the case, especially when it comes to complementarity and other concepts introduced by Bohr (e.g., [31]). 

These considerations extend to the concept of theory, again, *as understood here*, as other definitions of this concept are possible: a theory is an organized (conceptually, logically, or otherwise) assemblage of concepts, as just defined. Every theory, again, has its history in preceding theories and can change by modifying its concepts or the relationships among them. A viable physical theory must, however, relate, by means of logically consistent and experimentally verifiable propositions (possibly probabilistic or statistical in nature), to the multiplicity of phenomena or objects that are assumed to form the reality considered by this theory. This relation, in modern physics usually by means of mathematical models (defined below), might be representational, and derive its predictive capacity, essential for any physical theory, from this representation, or be merely predictive, possibly only probabilistically or statistically predictive. I refer to both phenomena and objects, because, as Kant realized, they are not the same even in classical mechanics, which deals with individual classical objects or sufficiently small classical systems. However, classical objects, say, planets moving around the Sun, and our phenomenal representation of them could be treated as the same for all practical purposes. This is because our observational interference could, in principle, be neglected or compensated for, thus allowing us to consider this behavior independently, a circumstance that does not appear to be expressly noted by Kant but that is crucial to Bohr, because this is no longer the case in quantum physics [32,33]. Doing so was assumed to be possible, at least in principle, in the case of all classical physical objects, even when they were not or even could not be observed, as in the case of atoms or molecules in the kinetic theory of gases.

Quantum phenomena put this assumption into question. Defined by the effects of the interactions between quantum objects and measuring instruments, quantum *phenomena* are observable in the same way as are classical physical objects and could be treated as classical objects. By contrast, the “uncontrollable” (quantum) nature of these interactions precludes any observation and, in RWR-type interpretations, an inferential reconstitution of the independent behavior of quantum *objects* [34,35]. Nobody has ever observed a moving electron or photon as such, independently, to the degree that the concept of motion, as opposed to a change of a state, ultimately applies to them, or any kind of quantum object. It is only possible to observe traces, such as spots on photographic plates, left by their interactions with measuring instruments. This still allows for a spectrum of assumptions concerning quantum objects and their behavior, beginning with the assumption of the existence of such objects (or what is so idealized), inferred from these traces. 

The present interpretation, while assuming this existence on the basis of these effects and their particular character (not found in classical physics), places quantum objects and behavior beyond conception, which I shall term “the strong RWR view,” rather than only representation, which I shall term “the weak RWR view.” The strong RWR view is a radical position. Not all interpretations in the spirit of Copenhagen go that far. Thus, while there are indications that Bohr, especially in the ultimate version of his interpretation (my primary focus here), might have agreed with this view (e.g., [35]), he never expressly stated so. One could assume the possibility of a mathematical representation of quantum objects and behavior in the absence of a physical conception of them. While Bohr’s and the present view exclude this possibility, Heisenberg was open to it in his later thinking (e.g., [36]).

The history of a theory is accompanied by the history of its interpretations. The history of QM, in particular, has been shaped by a seemingly uncontainable proliferation of, sometimes conflicting, interpretations. It is not possible to survey these interpretations here. Each rubric on by now a long list (e.g., the Copenhagen, the many-worlds, consistent-histories, modal, relational, transcendental-pragmatist, and so forth) contains different versions. The literature dealing with each interpretation is immense. Standard reference sources would list and summarize most common rubrics. Although often implicit, an interpretation is essential for establishing the relationships between a theory and the phenomena or objects it considers, essential to any theory. This is customarily done by means of mathematical models.

I define a *mathematical* model *in physics* as a mathematical structure or a set of mathematical structures that enables such relationships (the concept of models in mathematics or mathematical logic is a separate subject, put aside here). As that of theory and other major concepts discussed here, the concept of a mathematical model or model, in the first place, has a long history, which is also a history of diverse definitions, and literature on the subject is extensive as well. It is not my aim to discuss the subject as such or engage with this literature, which would be difficult within the scope of this article. The present concept of a mathematical model, while relatively open, is sufficient to accommodate those models that I shall consider. A more detailed discussion of the present view of mathematical models is given [37,38] and of modeling in general, on the lines of analytic philosophy of science, in [39,40]. One must also keep in mind the difference between a mathematical model *of* a theory (a concept especially important in mathematical logic or the philosophy of mathematics) and a mathematical model *used by* a theory, with which I am concerned here. Mathematical models used in physics may be geometrical (as in general relativity, for example) or algebraic, as in QM and QFT, although geometrical models contain algebraic elements. (The geometrical aspects of algebraic models are a more complex matter considered below.) The relationships between a model and the objects or phenomena considered may be representational. In this case the elements of a model and relations among them would correspond or map the elements of reality and the relations among them and relate the theory to reality by means of this mathematical representation. The predictive capacity of the theory, essential for any theory, would then derive this representation. The mathematical models used in classical mechanics or relativity are examples of such models. Models may, however, also be strictly predictive, without being representational, as are the mathematical models used in QM or QFT, in RWR-type interpretations, the predictions of which are, moreover, probabilistic or statistical, again, even in the case of elementary individual quantum objects and behavior. An interpretation of a given theory is, thus, always an interpretation of how the mathematical model or models used by it relate to the phenomena or objects considered. A theory may, however, involve other interpretive aspects, defined by its concepts. For example, part of Bohr’s and, following Bohr, the present interpretation is a particular (interpretive) concept of measuring instruments used in quantum physics. According to this concept, the observable parts of measuring instruments are described my means of classical physics, while these instruments also have quantum parts, through which they interact with quantum objects, an interaction “irreversibly amplified” to what is observed in measuring instruments [41]. Placing quantum objects and behavior beyond representation or even conception is another interpretative feature, in the second case without giving quantum objects and behavior concepts. 

Rigorously, a different interpretation of a given mathematical model defines a different theory in the present definition of a theory, because this interpretation may involve specific concepts, such as the ones just mentioned in the case of the interpretation adopted here, which may not be shared by other interpretations, even those in the spirit of Copenhagen. For simplicity, however, I shall speak of the corresponding interpretation of the theory itself containing a given mathematical model, interpreted by this theory, say, of one or another interpretation of QM. Thus, initially, Heisenberg’s and Schrödinger’s *versions of the formalism* appeared as two different mathematical models, giving the same predictions. They were also accompanied by *two different theories*, initially designated quantum mechanics and wave mechanics, the first strictly algebraic and the second geometrical, by virtue of conceiving quantum-level reality as a continuous wave-like process, each theory, moreover, given different interpretations at the time (actually, interpreting either theory posed major difficulties then). These two models were quickly proven to be mathematically equivalent (there are several proofs, all of which involve additional assumptions and complexities, the most general one given by the Stone-von Neumann theorem), which allowed one to unify the mathematical model of QM, ultimately in terms of its Hilbert-space formalism, with some yet more abstract versions added later. By contrast, the two theories—quantum mechanics (underlying the model of matrix mechanics) and wave mechanics—which were based in two different sets of concepts remained different. While Schrödinger’s theory, based in the idea of a wave-like ultimate reality, had receded by the late 1920s, as Schrödinger’s wave function received an interpretation as a tool for predicting probabilities, rather than representing any physical process, this theory has never been entirely abandoned.

I now turn to the concept of reality, which I shall approach via Bohr’s elaboration, partially cited above, concerning the epistemological situation that the concept of complementarity reflects [29]:

The renunciation of the ideal of causality in atomic physics which has been forced on us is founded logically only on our not being any longer in a position to speak of the autonomous behavior of a physical object, due to the unavoidable interaction between the object and the measuring instruments which [interaction] in principle cannot be taken into account, if these instruments according to their purpose shall allow the unambiguous use of the concepts necessary for the description of experience. In the last resort an artificial word like “complementarity” which does not belong to our daily concepts serves only briefly to remind us of the epistemological situation here encountered, which at least in physics is of an entirely novel character.

It follows that “our not being any longer in a position to speak of the autonomous behavior of a physical object,” demands “a radical revision of our attitude toward the problem of physical reality,” ultimately depriving us of realism [42]. “The renunciation of the [classical] ideal of causality,” invoked by Bohr, is automatic, because, as I explain below, causality requires realism [42].

I shall now introduce a concept of reality that permits this revision. This concept itself is very general and is, arguably, in accord with most, even if not all (which would be impossible), currently available concepts of reality in realism and nonrealism, which would, respectively, assume this reality to be representable or at least conceivable and to be beyond representation or even conception. By *reality* I refer to that which exists or is assumed to exist, without making any claim concerning the *nature* of this existence, which thus may be placed beyond representation or even conception. I understand existence as a capacity to have effects on the world with which we interact and that, because it exists, has such effects upon itself. To ascertain such effects entails representation of these effects, but not necessarily of how they come about, which implies that a given theory might assume different levels of reality, some allowing for a representation or at least conceptions and others not.

In physics, the primary reality considered is that of matter, including radiation, generally governed by the concept of field, classical or quantum. The idea of matter is still a product of thought, which, however, is customarily assumed to be a product of the material processes in the brain, and thus of matter. Matter is commonly, but not always (although exceptions are rare), assumed to exist independently, and to have existed when we did not exist and to continue to exist when we will no longer exist, which may be seen as defining the independent existence of matter. This view is upheld in the RWR-type interpretations of QM, but in the absence of a representation or even conception of the character of this existence, for example, as either discrete or continuous. Discreteness only pertains to quantum phenomena, observed in measuring instruments, while continuity has no physical significance at all. It is only a feature of the formalism of QM, which, while mathematically continuous, relates to discrete phenomena by predicting the probabilities or statistics of their occurrence.

Physical theories prior to quantum theory have been realist theories, usually *representational* realist theories. Such theories aim to represent the corresponding objects and their behavior by mathematical models, assumed to idealize how nature works, an assumption sometimes referred to as “scientific realism.” More exactly, as noted earlier, such a theory is a representation that is then realized by a mathematical model, which mathematically represents the reality considered. Thus, classical mechanics (used in dealing with elemental individual objects and small classical systems), classical statistical mechanics (used in dealing, statistically, with large classical systems), or chaos theory (used in dealing with classical systems that exhibit a highly nonlinear behavior) are all realist theories, as concerns the ultimate reality they consider. While classical statistical mechanics does not represent the overall behavior of the systems considered because their great mechanical complexity prevents such a representation, it assumes that the individual constituents of these systems are represented by classical mechanics. As indicated earlier, the status of these theories as realist could be questioned, on Kantian lines, even in the case of classical mechanics, where the representational idealizations used are more in accord with our phenomenal experience, which is only partially the case in relativity. However, all these cases still allow for viable idealized realist and (classically) causal models.

One could also define another type of realism, which is not representational. This realism encompasses theories that presuppose an independent structure of reality governing the behavior of the ultimate objects these theories consider, while allowing that this architecture cannot be represented, even ideally, either at a given moment in history or perhaps ever, but if so, only due to practical epistemological limitations. In the first eventuality, a theory that is merely predictive may be accepted for lack of a realist alternative, but under the assumption that a future theory will do better, in particular as a representational realist theory. Einstein adopted this view toward QM, which he expected to be eventually replaced by such a theory.

The assumption of realism of either type is abandoned or even precluded in reality-without-realism (RWR) type interpretations of quantum phenomena and QM, beginning with that of Bohr. In such interpretations, the mathematical model of QM, defined by its mathematical formalism, becomes a strictly probabilistically or statistically predictive, rather than *deterministic*, model, even in considering elementary individual quantum objects and processes, which form quantum-level reality, while suspending or even precluding a representation and possibly a conception of this reality, and an assumption that this reality is *causal*, or classically causal. I distinguish “causality” and “determinism.” By classical causality I refer, ontologically, to the conception that the state of the system considered is determined at all future moments of time, once it is determined at a given moment of time, and by determinism, epistemologically, to the possibility of predicting the outcomes of such processes ideally exactly. This conception of causality has defined modern classical physics since Descartes, Galileo, and Newton, and philosophy beginning at least with Plato. As will be seen, causality may be defined differently, first, in a relativistic or local sense and, second, in a quantum-theoretical probabilistic sense. The probabilistic or statistical character of quantum predictions must, however, be maintained by realist interpretations of QM or alternative theories (such as Bohmian mechanics), to accord with quantum experiments, where only probabilistic or statistical predictions are possible. This is because the repetition of identically prepared experiments in general leads to different outcomes, and unlike in classical physics, this difference cannot be diminished beyond the limit defined by Planck’s constant, *h*, by improving the capacity of our measuring instruments, as manifested in the uncertainty relations, which would remain valid even if we had perfect instruments.

RWR-type interpretations do assume the concept of *reality*, defined as that which is assumed to exist, without, in contrast to realist theories, making any claims concerning the *character* of this existence, which is what makes this concept of *reality* that of “reality *without* realism” [43,44]. The existence of quantum objects or something that leads to this idealization (it is still an idealization) is inferred from the totality of effects they have on world we observed, specifically on experimental technology, without making claims concerning their independent behavior. Such interpretations place quantum objects and processes either beyond representation, the weak RWR view, or more radically, beyond conception, the strong RWR view, which I adopt here. As I said, Heisenberg at the time of his discovery and Bohr at nearly all stages of his thinking held at least a weak RWR view, with Bohr eventually moving closer to the strong RWR view, while Heisenberg eventually adopted a form of mathematical realism. In 1927, Bohr briefly and ambivalently entertained the idea that independent quantum behavior and thus the ultimate nature of quantum reality could be represented, moreover, causally, by the mathematical formalism of QM, while indeterminism was introduced by measurement [45]. Bohr’s ambivalence was due to the fact that one deals with the formalism over **C**, which is difficult to associate with physical representation and the fact that Schrödinger’s wave equation in fact applied to the coordinate and not a real space: “The symbolic character of Schrödinger’s method appears not only from the circumstance that its simplicity, similarly to that of the matrix theory, depends essentially upon the use of imaginary arithmetic quantities. But above all there can be no question of an immediate connection with our ordinary conceptions because the ‘geometrical’ problem represented by the wave equation is associated with the so-called co-ordinate space, the number of dimensions of which is equal to the number of degrees of freedom of the system, and, hence, in general greater than the number of dimensions of ordinary space” [46]. In any event, Bohr quickly abandoned the view that independent quantum behavior is represented by QM, under the impact of his exchanges with Einstein. However, championed by both Dirac’s and von Neumann’s influential books [47,48], this view has persisted and remains common [49]. 

Although Kant’s philosophy may be seen as an important precursor the RWR view, the strong RWR view is manifestly more radical than Kant’s view of noumena or things-in-themselves vis-à-vis phenomena or appearances formed in our minds. According to Kant, while noumena are unknowable, they are still in principle conceivable, especially when one’s thinking is helped by what he calls “Reason” [*Vernunft*], a higher faculty than “Understanding” [*Verstand*], which only concerns phenomena, although there is no guarantee, even for Reason, that this conception is correct [50]. Even the weak RWR view is still more radical than that of Kant, because, while a conception of quantum objects and behavior is in principle possible, it cannot be unambiguously used in considering quantum phenomena, at least as things stand now. I am not saying that the strong RWR view is physically necessary, but only that it is interpretively possible. There does not appear to be any experimental data that would compel one to prefer either the strong or the weak RWR view, or to definitively claim for either anything beyond its consistency or effectiveness. These views are, however, different philosophically because they reflect different limits that nature allows our thought in reaching its ultimate constitution.

Two qualifications are in order. First, one could, in principle, see the claim concerning merely the existence or reality of something to which a theory can relate without representing it as a form of realism. This use of the term realism is sometimes found in advocating interpretations of QM that are nonrealist in the present sense (e.g., [51,52,53]), although none of these works adopted the strong RWR view. However, the present definition of realism or, similarly, ontology is more in accord with most understandings of realism, representational or nonrepresentational, including in considering quantum theory in physics and the philosophy of physics. Secondly, the present argument does not aim to deny realism even in the present sense, still a generally preferred view.

It could be assumed that *something* “happens” between observations, as manifested in changes that we observed in the instruments used, such as a discrete change of the energy, a “quantum jump,” of an electron in an atom (statistical as any claim concerning such changes may be), if one keeps in mind the provisional nature of such concepts as “happens.” According to Heisenberg [54]:

There is no description of what happens to the system between the initial observation and the next measurement. … The demand to “describe what happens” in the quantum-theoretical process between two successive observations is a contradiction in adjecto, since the word “describe” [or “represent”] refers to the use of classical concepts, while these concepts cannot be applied in the space between the observations; they can only be applied at the points of observation.

The same, it follows, must apply to the word “happen” or any word we use, and we must use words and concepts associated to them, even when we try to restrict ourselves to mathematics as much as possible. There can be no physics without language, but quantum physics imposes new limitations on using it. Heisenberg adds later in the same book: “But the problems of language are really serious. We wish to speak in some way about the structure of the atoms and not only about ‘facts’—the latter being, for instance, the black spots on a photographic plate or the water droplets in a cloud chamber. But we cannot speak about the atoms in ordinary language” [55].

On the other hand, as Heisenberg noted on an earlier occasion, mathematics is, “fortunately,” free from the limitations of daily language and concepts, fortunately because one could take advantage of this freedom in creating QM, doing which might not even have been possible otherwise [56]. Mathematics, especially algebra (geometry is more connected to our phenomenal intuition of spatiality), also allows to circumvent the limits our phenomenal, representational intuition, also involving visualization, sometimes used, including by Bohr, to translate the German word for intuition, *Anschaulichkeit*. Bohr often spoke of quantum objects and behavior as beyond altogether beyond visualization, although ultimately for him both were beyond any representation, including mathematical one (e.g., [57,58]). As free from these limitations of language and ordinary, or philosophical or even physical concepts, mathematics could be assumed to represent quantum-level reality, as Heisenberg eventually came to believe. *Physics and Philosophy* and his other later writings do give mathematics at least some capacity to do so, still in algebraic terms, to the point of defining, following Wigner [59], elementary particles themselves as representations of symmetry groups. However, while crucial in QM or, even more so, in QFT, the role of symmetry need not depend on realism, physical or mathematical, because symmetry groups can be viewed as part of the probabilistically or statistically predictive machinery of QM and QFT. There are ontological and, specifically, geometrical symmetries, for example, those embedded in conservation laws by Noether’s theorems, which apply in quantum theory, where, however, they are manifested at the macro level of measuring instruments, described classically. The concept of group is, however, algebraic, even when used, on realist lines, in classical physics or relativity, or of course in geometry or topology. In any event, a form of mathematical (algebraic) realism advocated by Heisenberg in his later works appears to exclude the application of representational language or concepts apart from mathematical ones to the ultimate constitution of reality [60]. On the other hand, as his argument in his paper introducing QM [3] (discussed in Section 4) suggests, at the time of his discovery Heisenberg appears to have seen mathematics’ freedom from these limitations, while crucial for QM and even making its invention possible, in terms of its probabilistically predictive rather than representational capacity, a view held by Bohr, a least from 1928 on. For Bohr, again, a mathematical representation, even if not a conception of quantum objects and behavior was “*in principle* excluded,” along with a physical one, at least as things stands now [35].

“As things stand now” is an important qualification, equally applicable to the strong RWR view, even though it might appear otherwise, given that this view precludes any conception of the ultimate reality not only now but also ever, by placing it beyond thought altogether. The qualification “as things stand now” still applies because a return to realism is possible, either on experimental or theoretical grounds even for those who hold this view. This return may take place because quantum theory, as currently constituted (QM, QFT, and QTFD), may be replaced by an alternative theory that allows for or requires a realist interpretation, or because RWR-type interpretations, either of the weak or the strong type, may become obsolete, even for those who hold this view, with quantum theory in place in its present form. As things stand now, either RWR view is interpretively possible. It is also possible, however, that the RWR view, in either weak or strong version, will remain part of our future fundamental theories, as the development of QFT theory appears to indicate. QFT has been open to the RWR-type view and the corresponding interpretation from its inception with Dirac until now and was used, specifically by Bohr, in support of this view (e.g., [61,62]). It also conforms to the QPA principle, even though it may appear and in some respects is more geometrical than QM, given the role of certain geometrical concepts, such as symmetries, in quantum theory, especially in QED, such as gauge symmetry, introduced by Weyl, initially in his (failed) attempt to (geometrically) unify general relativity and electromagnetism. In QED or QFT in general, however, the “geometry” of gauge symmetries is symbolic, and their real significance is the invariance under the corresponding gauge groups, say, as applies to the “phases” of electrons (“phase” being a symbolic concept, which physically relates to probabilities), is just part of the algebra of QFT theory which ultimately relates to the probabilities or statistics of experiments. As I said, there are spatial (or temporal) geometrical symmetries, such as those involved, by Noether’s theorems, in conservation laws, or still others, which are used in QFT, or QM, but these are only manifested at macro-levels. Feynman’s path integrals, which suggest trajectories and thus geometry, can be seen along the same algebraic lines (as part of the algebraic probabilistic machinery of QFT), especially given that these “paths” do not refer to the actual motion of particles, although the subject, admittedly, needs more discussion, which cannot be pursued here. 

It is also true that we use visual tools, such as Feynman’s diagrams. Enormously helpful as they are, however, Feynman’s diagrams are only *diagrams*, heuristic devices: they do not represent the quantum processes to which they refer, even if one holds that these processes are representable. The role of Feynman’s diagrams may be said to represent the predictive workings of the *formalism* of QED or QFT and thus to help one to work with this formalism in order to make probabilistic or statistical predictions concerning the outcomes of the experiments these diagrams are connected to, predictions only possible rigorously, numerically, by means of the algebra of QED or QFT. This, however, is quite different from representing quantum behavior itself. In sum, while there are additional nuances as concerns their difference, the formalism of QFT is algebraic in the same way as is that of QM, and the QPA and (interpretively) RWR principles combine analogously.

I now turn to the question of causality. As noted, RWR-type interpretations make the absence of classical causality nearly automatic. This absence is strictly automatic if one adopts the strong RWR view, which places the ultimate nature of reality beyond conception, because the assumption that this nature is classically causal would imply at least a partial conception of this reality. However, even if one adopts the weak RWR view, which only precludes a representation of this reality, classical causality is still difficult to maintain in considering quantum phenomena. This is because to do so one requires a degree of representation, analogous to that found in classical physics, that appears to be prevented, in particular, by the uncertainty relations (which are independent of QM). Schrödinger expressed this difficulty, while disparaging QM, or at least the spirit of Copenhagen, as “the doctrine born of distress,” in his cat-paradox paper: “if a classical state does not exist at any moment, it can hardly change causally,” where a classical state is defined by the (ideally) exact position and momentum of an object at any moment of time [63]. According to Bohr, who did not share Schrödinger’s reservations [64]:

It is most important to realize that the recourse to probability laws under such circumstances is essentially different in aim from the familiar application of statistical considerations as practical means of accounting for the properties of mechanical systems of great structural complexity. In fact, in quantum physics we are presented not with intricacies of this kind, but with the inability of the classical frame of concepts to comprise the peculiar feature[s] of the elementary [quantum] processes.

While “the classical frame of concepts” might refer to those of classical physics, Bohr might have been here closer to the strong RWR view, because at this stage of his thinking, he argues that all our representational concepts (“object” and “process,” among them) are classical, possibly apart from purely mathematical concepts, considered as entirely divorced from any phenomenal representation. Indeed, according to Wittgenstein, we may be unable to conceive of a process that is not causal [65]. Complementarity is different: While it has representational aspects, referring to phenomena observed in measuring instruments and thus to our experience, it does not represent the independent properties and behavior of quantum objects, but is instead designed to deal with a lack of this representation.

The question of causality is, however, a subtle matter, especially given that one can define concepts of causality that are not classical, and it merits a further discussion. First, I shall consider the concepts of indeterminacy, randomness, chance, and probability, again, as I understand them, because they, too, can be defined otherwise. In the present definition, indeterminacy or chance is a more general category, while randomness will refer to a most radical form of indeterminacy, when even a probability is not and cannot be assigned to a possible future event. Indeterminacy (including randomness) and chance may be understood as different from each other as well. These differences are, however, not germane in the present context, and I shall for convenience only refer to indeterminacy. An indeterminate, including random, event may or may not result from some underlying classical causal processes, whether this process is accessible to us or not. The first eventuality defines classical indeterminacy or randomness, conceived as ultimately underlain by a hidden classically causal architecture; the second irreducible indeterminacy and randomness. The ontological validity of an application of the latter cannot be guaranteed: it is impossible to ascertain that an apparently indeterminate or random sequence is in fact indeterminate or random, and there is no mathematical proof that any sequence is [66]. This concept is an assumption that may only be practically justified insofar as an effective theory or interpretation is developed.

As explained, factually, quantum phenomena only preclude determinism, because identically prepared quantum experiments, as concerns the state of measuring instruments, in general lead to different outcomes. Only the statistics of multiple identically prepared experiments are repeatable. It would be difficult, if not impossible, to do science without being able to reproduce at least the statistical data. The lack of classical causality or of realism in the RWR-type interpretations of quantum phenomena and QM are *interpretive inferences* from this situation and additional features such as correlations, the uncertainty relations, or complementarity. Such interpretations, again, do not exclude the possibility of causal or realist interpretations of QM, or alternative causal or realist quantum theories, such as Bohmian mechanics (which is, however, nonlocal), or theories defined by deeper underlying causal dynamics, which makes QM an “emergent” theory, such as A. Khrennikov’s “pre-quantum classical statistical field theory” [44,67].

Although sometimes glossed over, the difference between probability and statistics is important in quantum theory. I would like to briefly comment on this difference and on the role of probability and statistics in quantum theory more generally from the RWR-type perspective. My remarks cannot do justice to the subject, extensively considered in literature (e.g., [5,68]). They are only aimed to address those points that are especially relevant for my argument. “Probabilistic” commonly refers to our estimates of the probabilities of either individual or collective events, such as that of a coin toss or of finding a quantum object in a given region of space. “Statistical” refers to our estimates concerning the outcomes of identical or similar experiments, such as that of multiple coin-tosses or repeated identically prepared experiments with quantum objects, or to the average behavior of certain objects or systems. The standard use of the term “quantum statistics” refers to the behavior of large multiplicities of identical quantum objects, such as electrons and photons, which behave differently, in accordance with, respectively, the Fermi-Dirac and the Bose-Einstein statistics, for identical particles with, respectively, half-integer and integer spin. The Bayesian understanding defines probability as a degree of belief concerning a possible occurrence of an individual event on the basis of the relevant information we possess (e.g., [69] or, in a different version [70]). This makes the probabilistic estimates, generally, subjective, although there may be agreement (possibly among a large number of individuals) concerning such estimates. The frequentist understanding, also referred to as “frequentist *statistics*,” defines probability in terms of sample data by emphasis on the frequency or proportion of these data, which is considered more objective. In quantum physics, as noted, exact predictions are in general impossible even in dealing with elemental individual processes and events. This situation could, however, be interpreted either on Bayesian lines, under the assumption that a probability could be assigned to individual quantum events, or on frequentist lines, under the assumption that each individual effect is strictly random. A prominent recent example of a nonrealist Bayesian approach is Quantum Bayesianism, QBism, which, however, contains other philosophical dimensions (e.g., [52]). Although most of its argument would apply if one adopts a Bayesian view, this article adopts the frequentist, RWR-type, view, considered in detail in [43,44]. Bohr and Heisenberg appear to have been inclined to a statistical view of the type adopted here [71].

A brief qualification might be in order concerning two different uses of the statistical just mentioned, concerning, respectively, multiple repeated experiment and the average behavior of large system. One can make a *probabilistic* estimate for an event of finding an electron gas occupying less than a given volume, similarly to that of finding a quantum object in a given region of space. (In fact, this is true for events pertaining to classical statistical systems.) My point here, however, is that, unlike in classical mechanics, in QM we are dealing with randomness or probabilities even in considering events associated with elemental individual objects, such as electrons, rather than with large (“statistical”) multiplicities, and that such individual events can be interpreted on either Bayesian or statistical lines, in the latter case, under the assumption that such individual events are strictly random. These two interpretations would also distinguish individual events, such as those of finding an electron gas occupying less than a given volume. In the statistical interpretation in the present sense we would need to repeat this experiment many times to establish these statistics, while no probability is, in general, assigned to a given event.

Finally, probability introduces an element of order into situations defined by the role of randomness in them and enables us to handle such situations better. In other words, probability or statistics is about the interplay of indeterminacy or randomness and order. This interplay takes on a unique significance in quantum physics, because of the existence of quantum correlations, such as the EPR or (as they are also known) EPR-Bell correlations, found in the experiments of the Einstein-Podolsky-Rosen (EPR) type and considered, in the case of discrete variables, in Bell’s and the Kochen-Specker theorems, and related findings. These correlations are a form of statistical order. They are properly predicted by QM, which is, thus, along with and responding to quantum phenomena themselves, as much about order as about indeterminacy or randomness, and, most crucially, about their unique combination in quantum physics. The correlations themselves are collective, statistical, and as such they would not depend on either interpretation, Bayesian or frequentist, of our predictions concerning the individual events involved. That, in certain circumstances, indeterminate or random individual events form statistically correlated and thus ordered multiplicities is, however, one of the greatest mysteries of quantum physics, which makes it as much about order as about indeterminacy and randomness, a statistically correlated order without an ontologically underlying classical order that merely cannot be accessed epistemologically.

I shall now consider two alternative conceptions of causality important for my argument. Thus, the term “causality” is often used in accordance with the requirements of special relativity, which restricts (classical) causes to those occurring in the backward (past) light cone of the event that is seen as an effect of this cause, while no event can be a cause of any event outside the forward (future) light cone of that event. In other words, no physical causes can propagate faster that the speed of light in a vacuum, *c*, which requirement also implies temporal locality. Technically, this requirement only *restricts* classical causality by a relativistic antecedence postulate, rather than precludes it, and relativity theory itself, special or general, is (locally) a classically causal and indeed deterministic theory. By contrast, while, as a probabilistic or statistical theory of quantum phenomena, QM, at least in RWR-type interpretations, lacks classical causality, its probabilistic or statistical predictions are consistent with both temporal and spatial locality, and hence the relativistic antecedence. The same is true in the case of QTFD or QFT, and QFT in its standard form conforms to special relativity (although there are nonrelativistic versions of QFT). Thus, the compatibility with relativistic or, more generally, locality requirements would be maintained insofar as an already performed experiment determines, probabilistically or (if repeated many times) statistically, a possible outcome of a future experiment, without assuming classical causality. Determinism is, again, precluded on experimental grounds. Whatever *actually happens* is defined by spatially and temporally local factors, although the probabilistic or statistical *predictions*, could concern distant events, sometimes, as in the EPR-type experiments, without previously performing a measurement on the object concerning which one makes a prediction [34,72].

Relativistic causality is, thus, a manifestation of a more general concept or principle, that of locality. This principle states that no instantaneous transmission of physical influences between spatially separated physical systems (“action at a distance”) is allowed or that physical systems can only be physically influenced by their immediate environment. It is true that locality is a spatial or spatiotemporal concept (there is a temporal locality, which precludes, for example, retroaction in time and backward in time causality), which makes it geometrical. However, although it is an effect of the ultimate reality (which is, in RWR-type interpretations, beyond representation or even conception), locality manifests itself only classically, in what is observed in measuring instruments, where the geometrical considerations are fully applicable. Events, as we observe them, do happen in space and time: otherwise they could not be observed. In general, some geometrical considerations remain unavoidable in algebraic physical theories, such as QM, in part, again, because these theories must relate, at least in term predictions to phenomena observed in space and time. 

Locality of quantum phenomena and QM was at stake in the Bohr-Einstein debate from its inception in the late 1920s, but especially following EPR’s paper [34,72]. As Bohr argued in his reply to EPR’s paper (which argued that QM is either incomplete or else nonlocal), standard QM avoids nonlocality, at least in the RWR-type interpretations of the theory and quantum phenomena themselves, even though, as I said, under certain circumstances, such as those of the EPR-type experiments, QM can make *predictions* concerning the state of spatially separated systems, while, crucially to Bohr’s argument, the physical circumstances of making these predictions and verifying them are local [73]. The question of the locality of QM or quantum phenomena is, however, a matter of much debate and controversy, especially in the wake of the Bell and Kochen-Specker theorems and related findings, as well as numerous experiments dealing with correlations, beginning with, most famously, those by Aspect [74], based on Bohm’s version of the EPR experiments. These debates cannot be addressed within the scope of this article, and the literature dealing with these subjects is nearly as extensive as that on interpretations of QM (e.g., [75,76,77,78]). As in Bohr’s exchange with EPR, the question of the relationships between locality and realism, or a lack thereof, figures centrally in these debates and the findings just mentioned. 

Finally, I would like to propose the concept of quantum causality. I shall do so via Bohr’s concept of complementarity, which Bohr saw as a generalization of causality. Complementarity is defined by:

(a) a mutual exclusivity of certain phenomena, entities, or conceptions; and yet
(b) the possibility of considering each one of them separately at any given point; and
(c) the necessity of considering all of them at different moments for a comprehensive account of the totality of phenomena that one must consider in quantum physics.

Complementarity may be seen as a reflection of the fact that, in a radical departure from classical physics or relativity, the behavior of quantum objects of the same type, say, electrons, is not governed, individually or collectively, by the same physical law, in all possible contexts, specifically in complementary contexts. Speaking of “*physical* law” in this connection requires caution, because, in Bohr’s interpretation, there is no physical law representing this behavior, not even a probabilistic law if one adopts a statistical, rather than a Bayesian, view of the individual quantum behavior. The behavior of quantum objects leads to mutually incompatible observable physical effects in complementary contexts. On the other hand, the mathematical formalism of QM offers correct probabilistic or statistical predictions (no other predictions are possible) of quantum phenomena *in all contexts*.

It follows, especially if one adopts an RWR-type of interpretation, that the nature of both experimental and theoretical physics changes. Experimentally we no longer track, as we do in classical physics or relativity, the independent behavior of the systems considered, track, in effect geometrically, what happens in any event. Instead we define what *will* happen in the experiments we perform, by *how* we experiment with nature by means of our experimental technology, even though and because we can only predict what will happen probabilistically or statistically. Thus, in the double-slit experiment, the two alternative setups of the experiment, whether we, respectively, can or cannot know, even in principle, through which slit each particle, say, an electron, passes, we obtain two different outcomes of the statistical distributions of the traces on the screen (with which each particle collides). Although sometimes associated with the “wave” behavior of quantum objects, the “interference pattern” observed in the second setup is a statistically ordered pattern of discrete traces left by the collisions between the particles and the screen. This is one of the reasons why Bohr avoided speaking of wave-particle complementarity, even though the latter is commonly used to illustrate Bohr’s concept. In Bohr’s view, quantum objects could not be represented either in terms of particles or in terms of waves, and the pattern of traces in questions were, again, only effects on the interactions between quantum objects and measuring instruments. Or, in effect equivalently to the double-slit experiments, we can set up our apparatus so as to measure and correspondingly predict, again, probabilistically or statistically, either the position or the momentum of a given quantum object, but never both together. Either case requires a separate experiment, incompatible with the other, rather than representing “the arbitrarily picking out of different elements of physical reality at the cost of other such elements [all pertaining to the same quantum object]” within the same physical situation, by tracking either one of its aspects or the other, as we do in classical mechanics [79]. There, this is possible because we can, at least in principle, assign simultaneously both quantities within the same experimental arrangement. In quantum physics, we cannot. Quantum physics, again, changes what experiments do: they *define* what will happen, rather than follow what is bound to happen in accordance with classical causality. It is true that we can sometimes define by an experiment what will happen in classical physics. In this case, however, we can then observe the resulting process without affecting it by observation. This is not the case in quantum physics, because *any new observation* defines a new course of events. Only *some observations* do in classical physics. By the same token, at least in RWR-type interpretations, quantum theory only tells us possible things about the future, never about the past, which is only determined by measurements.

It is this probabilistic or statistical determination (which precludes classical causality but respects locality) of what can happen as a result of our conscious decision concerning which experiment to perform at a given moment in time, that defines what I call “quantum causality” [80]. Whatever is registered as a quantum event (providing the initial data) defines a possible set of, probabilistically or statistically, predictable future events, outcomes of possible future experiments. This definition is in accord with recent views causality in quantum information theory (e.g., [81,82,83]), except that it is linked to our conscious decision concerning experiments we perform, which is rarely considered. It is, however, this aspect of the situation that brings complementarity into play, because, in complementary situations, such a decision irrevocably rules out the possibility of making any predictions concerning certain other, complementary, events.

With these considerations in mind one can understand Bohr’s view of complementarity as a generalization of causality [84]. On the one hand, “our freedom of handling the measuring instruments, characteristic of the very idea of experiment” in all physics, our “free choice” concerning what kind of experiment we want to perform is essential to complementarity [79]. On the other hand, as against classical physics or relativity, implementing our decision concerning what we want to do will allow us to make only certain types of predictions and will exclude the possibility of certain other, *complementary*, types of predictions. Complementarity generalizes causality in the absence of classical causality and, in the first place, realism, because it defines which reality can and cannot be brought about by our decision concerning what experiment to perform. 

## 3. Geometry and Algebra in Physics and Beyond

However one assesses Einstein’s skeptical attitude toward the Heisenberg method, his view of it as algebraic was, I argue here, correct, and it helps one to better understand and contextualize Heisenberg’s discovery. Before I consider this discovery itself, however, a more proper examination of this characterization is necessary. First, I briefly revisit the basic understanding of geometry and algebra, starting with algebra, which is more straightforward, because geometry involves further complexities, in part by virtue of always containing algebraic components, while algebra can be free from geometry. Most generally, algebra is, as said, the mathematical formalization of the relationships between symbols, which makes it part of all mathematics, at least all modern mathematics (as the ancient Greek geometry only contained arithmetic). Thus, for example, both mathematical logic and calculus are forms of algebra in this general sense, even though, as fields, each contains its specificity. Another, narrower or field specific sense of algebra, that referring to algebraic structures such as groups or associative algebras, is equally crucial to quantum theory and its algebraic character. (As noted, symmetry groups are also crucial to geometry and geometrical physical theories, such as relativity, but there, too, they form part of the algebraic structures associated to geometrical and topological ones.) While the role of symmetry groups in quantum theory only became apparent a few years after Heisenberg’s discovery, it was well in place by the time of Einstein’s comment on the algebraic nature of the Heisenberg method in 1936. Third, algebra also refers to, and was born from, the study of algebraic equations, which still define a large part of the mathematical discipline of algebra. Forth, finally, the algebra of probability is central, at a fundamental, rather than, as in classical physics, merely practical, level in quantum theory. Heisenberg’s algebraic method and, following his work, quantum theory encompasses all these senses and aspects of algebra, some of which are shared with classical physics or relativity.

Geometry, generally defined here as the mathematical formalization of spatiality, especially (although not only) in terms of measurement is a more complex matter, because, on the one hand, this formalized spatiality still connects to our general phenomenal intuition, including visualization, of spatiality, and on the other, the role of mathematical formalization in geometry connects it to algebra. This connection allows one to generalize geometrical or topological objects far beyond anything our phenomenal intuition can access, especially by means of visualization. I would like now to address some of these complexities, as they pertain to my argument concerning the algebraic character of quantum theory. It would not be possible to treat this complex subject more generally.

Thus, we speak of and can rigorously define Hilbert *spaces* and their *geometry*, and in this sense, one could speak, as Dirac was reportedly fond of doing, of geometrical thinking in QM or QFT. I would argue, however, that these spaces and geometries are such primarily by extrapolation or, as it were, metaphorically, while, rigorously, they are essentially forms of *algebra*, as against the more standard forms of geometry, such as Euclidean or even non-Euclidean geometry, or differential geometry, used in general relativity, which are still more closely connected to our phenomenal intuition of spatiality. I would contend that this distinction is warranted and useful even though it is not absolute and there are gray areas. The geometries of these more conventional spaces are defined by certain algebraic properties and relations (beginning with metric), some of which can be used to define more abstract objects. Thus, these properties and relations define the *structure* of Hilbert spaces or, similarly, other mathematical “spaces,” such as those of projective geometries, abstract algebraic varieties, the spaces of noncommutative geometry, geometric groups, and so forth, in the absence of certain other, more conventionally spatial elements and structures, geometrical or topological, found in more conventional spatial objects, such as and in particular **R**^3^. The latter is the mathematical space that is the closest to our phenomenal spatiality, even though some of its mathematical (topological and geometrical) properties, beginning with continuity are far beyond our phenomenal intuition. As Weyl argued: “the conceptual world of mathematics is so foreign to what the intuitive continuum presents to us that the demand for coincidence between the two must be dismissed as absurd” [85]. “Coincidence” is, of course, not the same as “relation,” which might be unavoidable, at least in that it is difficult to think of continuity or spatially apart from one or other phenomenal intuitions of it. On the other hand, it is entirely possible to mathematically define continuous mathematical objects, such as **R**^3^, algebraically. This is why I speak of the extrapolated or metaphorical character of “space” and “geometry” of such objects as Hilbert spaces. Technically, **R**^3^ is a Hilbert space too, but it need not be considered as such, while Hilbert spaces considered in quantum theory do. This extrapolated or algebraic character arises because of the infinite-dimensional nature of some of those spaces or because they are defined over **C**, which appear irreducible in QM. While the Hilbert spaces involved are finite in the case of discrete variables in QTFD, they can have higher dimensions and are over **C**.

It is difficult to be certain, especially from reported statements, what Dirac exactly had in mind in his appeals to geometrical thinking in quantum theory. If, however, one is to judge by his writings, they appear to suggest that at stake were algebraic properties and relations modeled on those found in geometrical objects, as just explained. Indicatively, notwithstanding his insistence on the role of geometrical thinking in Dirac, Darigold’s analysis of this thinking shows precisely the significance of this type of algebra there. Thus, he says: “roughly, Dirac’s quantum mechanics could be said to be to ordinary mechanics what noncommutative geometry is to intuitive geometry” [86]. However, noncommutative geometry, the invention of which was in part inspired by the mathematics of QM, is a form of this kind of algebra [87,88].

In what sense, then, apart from being defined by such algebraic structures, may such spaces be seen as spaces, in particular, as relates to our phenomenal intuition, including visualization, from which QM departs, although, in Bohr’s or the present view it breaks with any representation of quantum objects and behavior, including a mathematical one? The subject is complex and it is far from sufficiently explored in cognitive psychology and related fields, an extensive research during recent decades notwithstanding, including as concerns cultural or technological (digital technology in particular) factors affecting our spatial thinking. Accordingly, it would be difficult to make any definitive claims. It does appear, however, that, these factors notwithstanding, our three-dimensional phenomenal intuition is shared by us cognitively and even neurologically in shaping our sense of spatiality. Part of this sense appear to be Euclidean, insofar as it corresponds to what is embodied in **R**^3^ (again, a mathematical concept), keeping in mind that the idea of empty space, apart from bodies of one kind or another defining or faming it, is an extrapolation, because we cannot have such a conception phenomenally or, as Leibniz argued against Newton, physically. We can have a mathematical conception of space itself. To what degree our phenomenal spatiality is Euclidean remains an open question, for example, in dealing with the visual perception of extent and perspective (e.g., [89,90,91]). 

It is nearly certain, however, that, when we visualize such algebraically defined spatial objects as Hilbert spaces or even more conventional geometrical spaces or geometries, once the number of dimensions is more than three, or for spaces of any dimensions defined over fields other than **R**, such as **C** or algebraic fields of finite characteristics, we visualize only three- (and even two-) dimensional configurations and supplement them by algebraic structures and intuitions. Feynman instructively explained this process in describing visual intuition in thinking about quantum objects [92]. Obviously, such anecdotal evidence hardly suffices for any definitive claim, but it appears to be in accord with some of the current neurological and cognitive-psychological research, as just mentioned, which suggests the dependence of our spatial intuition, including visualization, on two- and three-dimensional phenomenality.

As noted earlier, according to Bohr and Heisenberg, classical mechanics, a mathematical science of bodies and motion in space and time, is a mathematical refinement of our daily phenomenal thinking concerning space, time, and motion, no longer workable in quantum theory or, again, even in relativity in considering photons or motions with speed close to the speed of light in a vacuum, in which cases, however, the behavior of the objects considered can be properly handled algebraically, in quantum theory in probabilistic or statistical terms [56,93]. Accordingly, one could maintain the difference that I argue for between geometrical character of classical mechanics or (with qualifications) relativity and the algebraic character of quantum theory, especially in RWR-type representations of the latter. 

This is not to say that the geometrical or topological character of such objects disappears; quite the contrary, this character remains crucial on at least two counts. First, algebra has a special form that may be called “spatial algebra.” Selecting a good term poses some difficulties because such suitable terms as “geometric algebra” and “algebraic geometry” are already in use for designating, respectively, the Clifford algebra over a vector space with a quadratic form, and the study of algebraic varieties, defined as the solutions of the systems of polynomial equations. “Spatial algebra” arises from algebraic structures that *mathematically* define what are conventionally known as geometrical or topological objects, such as projective spaces or topological manifolds, and reflect their proximity to **R**^3^ and mathematical spatial objects that are close to our phenomenal intuition, and the geometry and topology associated with it. This proximity may be left behind in the rigorous mathematical treatment of such objects, beginning with **R**^3^. The same type of spatial algebra also defines objects, from projective and finite (topologically discrete) spaces to the infinite-dimensional spaces, where these connections to phenomenally visualizable spatial objects are only spatial algebraic, unless used by an extrapolation or metaphorically, as in the case of a projective space (a set of lines through the origin of a vector space, such as **R**^2^ in the case of projective plane, with projective curves defined algebraically, as algebraic varieties) or an infinite-dimensional Hilbert space (the points of which are typically square-integrable functions or infinite series). Spatial algebra is the algebraization of spatiality that makes it rigorously mathematical. 

At the same time, however, and this is the second count on which mathematical objects defined by spatial algebra retain their connections to geometrical and topological thinking, analogies with **R**^3^ continue to remain useful and even indispensable. Such analogies may be rigorous (and hence algebraic) or phenomenally intuitive or metaphorical. Thus, the analogues of the Pythagorean theorem or parallelogram law in Euclidean geometry, which holds in infinite-dimensional Hilbert spaces over either **R** or **C**, are important, including in applications to physics, such as QM. More generally, our thinking concerning geometrical and topological objects is not entirely translatable into algebra. This was well understood by Hilbert in his axiomatization of Euclidean geometry, even though this axiomatization had a spatial algebraic character, including in establishing an algebraic model (the field of complex numbers) for his system of axioms in order to prove its consistency [94]. 

One can gain a further insight into this situation by considering a related principle, “Think Geometrically, Prove Algebraically,” advanced by Tate, whose thinking bridged number theory and algebraic geometry in highly original and profound ways. The principle was introduced in the book (with Silverman), on “the rational points of elliptic curves.” The title-phrase itself combines algebra (“rational points”) and “geometry” (curves), and implies that geometry, at least beyond that of **R**^3^ and even there, requires algebra to be mathematically rigorous. According to Silverman and Tate [95]:

It is also possible to look at polynomial equations and their solutions in rings and fields other than **Z** or **Q** or **R** or **C**. For example, one might look at polynomial with coefficients in the finite field F*_p_* with *p* elements and ask for solutions whose coordinates are also in the field **F***_p_*_._ You may worry about your geometric intuitions in situations like this. How can one visualize points and curves and directions in **A**^2^ when the points of **A**^2^ are pairs (*x*, *y*) with *x*, *y* ∈ **F***_p_*? There are two answers to this question. The first and most reassuring is that you can continue to think of the usual Euclidean plane, i.e., **R**^2^, and most of your geometric intuitions concerning points and curves will still be true when you switch to coordinates in **F***_p_*. The second and more practical answer is that the affine and projective planes and affine and projective curves are defined *algebraically* in terms of ordered pairs (*r*, *s*) or homogeneous triples [*a*, *b*, *c*] without any reference to geometry. So in proving things one can work algebraically using coordinates, without worrying at all about geometrical intuitions. We might summarize this general philosophy as: *Think Geometrically*, *Prove Algebraically*”.

The affine and projective planes and curves can, in principle, be defined without any reference to ordinary language and concepts, which are more difficult and perhaps impossible to avoid in geometry, that is, in the kind of intuitive geometry they refer to, rather than spatial algebra that ultimately defines all geometry rigorously. Rigorously, in these cases, we think algebraically, too, by using spatial algebra, even if with the help of geometrical intuitions. It is true that a mathematician like Tate can develop and use intuition in dealing with discrete geometries as such, say, that of the Fano plane of order 2, which has the smallest number of points and lines (seven each). However, beyond the fact that they occur in the two-dimensional regular plane, the diagrammatic representations of even the Fano plane are still difficult to think of as other than spatial algebra, in this case, combinatorial in character. For the moment, while useful and even indispensable, our Euclidean intuitions are limited even when we deal with algebraic curves in the usual Euclidean plane, let alone in considering something like a Riemann surface as a curve over **C**, or curves in finite geometries, abstract algebraic varieties, Hilbert spaces, the spaces of noncommutative geometry, or geometric groups, a great example of the extension, by a reversal, of spatial algebra to conventionally algebraic objects as it is. This also means, as Tate must have been aware, that mathematical *thinking* concerning geometrical and topological objects cannot be reduced to those naïve intuitions. Silverman and Tate’s next example from differential calculus, that of finding a tangent line to a curve, confirms this point. The invention of calculus, an essentially algebraic form of mathematics, was not about proving things algebraically, as the standard of proof then was geometry, which compelled Newton to present his mechanics in terms of geometry in his 1687 *Principia*, in order, as he said, to assure a geometrical demonstration of his findings, also in the direct sense of showing something by means of visualization, rather than in terms of calculus [96]. Calculus was about *thinking* algebraically, as was especially manifested in Leibniz’s version, rather than about rigorous proofs. 

Einstein was, then, quite correct in characterizing the Heisenberg method as “algebraic.” The “geometry” of the Hilbert spaces of quantum theory only confirms the algebraic character of this method, especially manifested in his matrix or operator algebra. Still, a few additional qualifications are necessary for rigorously maintaining Einstein’s and the present view of the Heisenberg method as algebraic. First of all, in saying that “we must give up, in principle, the space-time continuum,” Einstein must have had in mind the spacetime continuum in representing, by means of the corresponding theory, the ultimate reality considered, and possibly in attributing the space-continuum to this reality. The idea that this reality may ultimately be discrete had been around for quite a while by then: it was, for example, proposed by Riemann as early as 1854, by way of a remarkable phrase “the reality underlying space,” thus potentially divorcing on proto-RWR lines reality and realism, at least a form of realism according to which our phenomenal or even mathematical representation of space was continuous [97]. The idea of the discrete nature of the ultimate reality has acquired new currency in view of QM and QFT, advocated by, among others, Heisenberg in the 1930s, and is still around.

One must also keep in mind the complexity of the algebra of QM or QFT, which involves objects that are not, in general, discontinuous, although certain key elements involved are no longer continuous functions, such as those in classical physics. Some continuous functions are retained, because the Hilbert spaces involved are those of such functions, considered as infinite-dimensional vectors in the case of continuous variables such as position and momentum, which variables themselves are represented by operators. These functions are those of complex (rather than, as in classical physics, real) variables and the vector spaces that they comprise or associated objects, such as operator algebras, have special properties, such as noncommutativity. Indeed, given that it deals with Hilbert spaces, QM or QFT involves mathematical objects whose continuity is denser than that of regular continua such as the (real number) spacetime continuum of classical physics or relativity. In contrast to these theories, however, the continuous and differential mathematics used in quantum theory, along with the discontinuous algebraic one, relates, in terms of probabilistic predictions, to the physical discontinuity defining quantum phenomena, which are discrete in relation to the observable spacetime continuum and to each other, while, at least in non-realist, RWR-type, interpretations, quantum objects and their behavior are not given any physical or mathematical representation—continuous or discontinuous (or mixed). Born and Jordan developed a differential calculus, “symbolic differentiation,” as they called it, for matrices used in quantum mechanics [8,98]. So did Dirac in his first paper on quantum mechanics [99]. In the style of Leibniz, this differentiation was defined algebraically by using the noncommutation rules. This quantum differentiation enables one to retain the differential equations of classical mechanics and their accompanying machinery, such as and in particular the Poisson bracket, while using new quantum variables. The quantum-mechanical analogue of the Poisson bracket is the expression $\frac{2\pi i}{h}\left(pq-qp\right)$, as Dirac was first to realize, with far-reaching implications for quantum mechanics. Dirac’s starting point, again, in the style of Leibniz, was the quantum-mechanical analogue of the rule for the differential of the product of two functions, which may be seen as a linear operator and which may be suitably algebraically quantized [99]. 

## 4. From Geometry to Algebra, with Heisenberg

For nearly a century since the publication of von Neumann’s seminal *The Mathematical Foundation of Quantum Mechanics* [48], the mathematical models of quantum theory—QM, QTFD, and QFT—have been commonly defined in terms of the Hilbert-space formalism, which remains dominant, even though there are other versions, such as C*-algebras and, more recently, the one based in category theory. Von Neumann’s aim was to give a proper mathematical grounding to the quantum-mechanical formalism, already developed by Heisenberg, Born, Jordan, Dirac, and others. By contrast, Heisenberg’s aim was to *find* a successful theory accounting for the behavior of the electrons in the atoms, which he accomplished with his new mathematical model, admittedly preliminary, but quickly developed into a more rigorous model, matrix mechanics, by Born and Jordan [8]. Dirac offered the most general version of the formalism before von Neumann, who thought that Dirac’s version lacked a proper mathematical rigor, in part because of Dirac’s use of delta function, not considered mathematically legitimate. Eventually, in the 1940s, delta function was given a proper definition, as the so-called “distribution,” a functional, by Schwartz, which legitimatized Dirac’s formalism mathematically. 

As stated from the outset, Heisenberg abandoned the project of representing the behavior of electrons in atoms. It is true that he only thought that such a representation was unlikely to be achieved at the time, as opposed to arguing, as Bohr did in the 1930s, that such a representation and even an analysis, if not conception, of quantum behavior was “*in principle* excluded” [35]. Still, Heisenberg’s was an audacious and radical move, which decisively shaped Bohr’s subsequent thinking, although Heisenberg’s thinking was in turn influenced by Bohr’s 1913 atomic theory. Bohr’s theory only partially abandoned the geometrical representation of quantum behavior in the case of “quantum jumps”—transitions between the stationary states of electrons, conceived in terms of electrons orbiting the nuclei. While Bohr’s theory, as developed by him and others had major successes, by the early 1920s it proved to be ultimately unsustainable. This failure compelled Heisenberg to renounce a geometrical representation of any quantum behavior in space and time, including that of stationary states in terms of orbits. This renunciation led him to his discovery of QM. There was no longer any algebraic representation of quantum objects and behavior either. 

Heisenberg’s approach was grounded in a set of fundamental principles, in part stemming from Bohr’s 1913 theory. As in the case of concepts, although one could sometimes surmise the meaning of the term “principle” from its use, it is, similarly to the term “concept,” rarely defined or explained in physical or even philosophical literature. His title notwithstanding, Heisenberg did not do so in his first book, *The Physical Principles of the Quantum Theory* [100] nor did Dirac, in his famous *Principles of Quantum Mechanics*, published in the same year [47]. Terms like “principle,” “postulate,” and “axiom,” are often used in physics somewhat indiscriminately, and it is difficult to entirely avoid overlapping between the concepts designated by these terms, or those designated as “laws,” especially because physical principles often derive from (or give rise to) postulates or laws. It may also be a matter of the functioning of these concepts. Thus, conservation laws are sometimes seen as conservation principles. For present purposes, I shall adopt the concept of principle from Einstein’s concept of a “principle theory,” which he introduced by way of juxtaposing this concept to that of a “constructive theory.” This concept corresponds to the use of principles by Bohr and Heisenberg, and by quantum-information theorists discussed in the next section. According to Einstein, constructive theories aim “to build up a picture of the more complex phenomena out of the materials of a relatively simple formal scheme from which they start out,” which, it follows, also make such theories realist. By contrast, principle theories “employ the analytic, not the synthetic, method. The elements which form their basis and starting point are not hypothetically constructed but empirically discovered ones, general characteristics of natural processes, principles that give rise to mathematically formulated criteria which the separate processes or the theoretical representations of them have to satisfy” [101]. I would add the following qualification, which is likely to have been accepted by Einstein: Principles are not empirically discovered but formulated on the basis of empirically established evidence. A principle theory may also be a constructive theory, but it need not be, and Heisenberg’s mechanics was not.

Heisenberg’s approach and then Bohr’s interpretation of QM were grounded in the following three principles (with Bohr’s principle or at least concept of complementarity added in 1927), which fit and even embody the “equation” *QUANTUMNESS* → *PROBABILITY* → *ALGEBRA* and the QPA principle:

(1) the principle of discreteness, the QD principle, according to which all observable quantum phenomena are individual and discrete in relation to each other, which is different from the discreteness of quantum objects;
(2) the principle of the probabilistic or statistical nature of quantum predictions, the QP/QS principle, which is maintained, in contrast to classical statistical physics, even in considering elemental individual quantum processes, and is accompanied by a special, nonadditive, character of quantum probabilities and rules, such as Born’s rule, for deriving them; and
(3) the correspondence principle, which, as initially understood by Bohr, required that the predictions of quantum theory must coincide with those of classical mechanics in the classical limit, but which was given by Heisenberg a form of “the mathematical correspondence principle,” requiring that the equations and variables of QM convert into those of classical mechanics in the classical limit.

To connect his formalism (defined over **C**) to the probabilities of outcomes of quantum experiments (which probabilities are real numbers), Heisenberg used a version of the Born rule in the special case of the transitions between stationary states, and not, as Born did, as universally applicable in QM. Referring to stationary states requires caution, because *stationary* only means that the electrons remained in their orbits with the same energy, were in the same “energy-state,” but would continuously change their position or their “position-state” along each orbit. On the other hand, the electrons would discontinuously, by quantum jumps, change their energy states, or their other states, by moving from one orbit to another. In Heisenberg, there were no longer orbits but only states and discontinuous transitions between states. As noted from the outset, one was no longer thinking, as in classical mechanics, in terms of predictions, even probabilistic predictions, concerning *a moving object*, say, an electron, free or orbiting the nucleus of an atom, but instead in terms of the probabilities of *transitions between the states of an electron*, transitions that were always discrete. This type of thinking emerged in Bohr’s 1913 theory in considering an electron’s transitions from one energy level to another, but, following Heisenberg, came to define quantum physics in general as a physics of predicting discrete transitions between states [12]. As Heisenberg said in his letter to Kronig (5 June 1925): “What I really like in this scheme is that one can really reduce *all interactions* between atoms and the external world… to transition probabilities” (cited in [102]).

Heisenberg’s scheme, thus, extended Bohr’s 1913 concept of discrete transitions or quantum jumps, which, unlike the orbital behavior of electrons in stationary states, had no mechanical or geometrical model, to all quantum behavior. The “concept of orbit,” as Heisenberg noted later, “had been somewhat doubtful from the beginning,” because of “the discrepancy between the calculated orbital frequency of the electrons and the frequency of the emitted radiation,” which had to be interpreted as a limitation to the concept of the electronic orbit” [103]. Accepting this discrepancy and, thus, dissociating these two types of frequencies was a revolutionary move on Bohr’s part, emphasized by Bohr himself: “How much the above considerations differ from an interpretation based on the ordinary electrodynamics is perhaps most clearly shown by the fact that we have been forced to assume that a system of electrons will absorb radiation of a frequency different from the frequency of vibration of electrons calculated in the ordinary way” [104]. Heisenberg rethought stationary states as just energy states, permitting no mechanical model or geometrical representation. There were, again, only transitions between quantum states (using the term “state” physically, rather than mathematically, as “a state vector”). 

By speaking of the “*interactions* between atoms and the external world,” Heisenberg’s statement in his letter to Kronig also suggests that QM, as he saw it, was about (predicting) these interactions, specifically with the measuring instruments involved, a view manifested in Heisenberg’s paper and adopted by Bohr. All that one could say about quantum objects and behavior could only concern their effects on measuring instruments, probabilistically or statistically predictable by QM. 

The mathematical correspondence principle motivated Heisenberg’s decision to retain the equations of classical mechanics, while, necessarily, introducing different variables, both, however, being now equally parts of his algebra. The correspondence with classical theory could still be maintained because new variables could be substituted for conventional classical variables (such as those of position and momentum) in the classical limit, as for large quantum numbers, when the electrons were far away from the nuclei and when classical concepts, such as orbits, could apply, thus also restoring the geometry of classical mechanics. The electrons’ behavior itself was still quantum and certain quantum effects, not observed in dealing with classical objects, could be observed, effects predictable only by the algebra of QM. The old quantum theory was defined by the strategy of retaining the variables of classical mechanics while adjusting the equations to achieve better predictions. Heisenberg’s reversal of this strategy was, thus, unexpected, and it required a radical change in the role these equations were to play. They no longer represented the motion of electrons, but served as the mathematical means enabling probabilistic or statistical predictions concerning effects of the interaction between electrons and measuring instruments. 

Heisenberg’s discovery was a remarkable achievement, ranked among the greatest in the history of physics. A detailed discussion of his derivation of QM is beyond my scope [105]. Several key features of his thinking are, however, worth commenting on, following [105], to further illustrate the algebraic nature of his thinking. Heisenberg’s new quantum variables were infinite unbounded matrices with complex elements. Their multiplication, which Heisenberg, who was famously unaware of the existence of matrix algebra and reinvented it, had to define, is in general not commutative. Essentially, these variables are operators in Hilbert spaces over **C**. Such mathematical objects had never been used in physics previously, and their noncommutative nature was, initially, questionable and even off-putting for some, including Heisenberg himself and Pauli [106]. In fact, while matrix algebra, in finite and infinite dimensions, was developed in mathematics by then, *unbounded* infinite matrices were not previously studied. As became apparent later, such matrices are necessary to derive the uncertainty relations for continuous variables. There are further details: for example, as unbounded self-adjoint operators, defined on infinite dimensional Hilbert spaces, these matrices do not form an algebra with respect to the composition as a noncommutative product, although some of them satisfy the canonical commutation relation. These details are, however, secondary. Most crucial was that the concept was used in a fundamentally new way. Heisenberg’s variables were algebraic entities enabling probabilistic or statistical predictions concerning *quantum phenomena*, observed in measuring instruments, without providing a mathematically idealized representation, geometrical or other, of the spacetime behavior of *quantum objects* responsible for these phenomena.

In this regard, although understandable historically, the term “observables” is misleading and is especially inadequate if one adopts a RWR-type view. As noted, Bohr saw the quantum-mechanical formalism as “symbolic” in the following, essentially algebraic, sense. While the mathematical symbols used in it appear, as variables, in the same equations as those used in classical mechanics, these symbols did not represent physical quantities pertaining to quantum objects themselves and their behavior, in the way such symbols do in classical mechanics. By the same token, the equations of QM, Schrödinger’s equation included, no longer functioned as equations of motion. Instead they are part of the probabilistic algebra of QM, enabling us to compile, in Schrödinger’s terms, “expectation-catalogs” concerning events observed in quantum phenomena, which, in RWR-type interpretations, gives Schrödinger’s equation an algebraic character along with a probabilistic one [63]. Schrödinger’s waves were symbolic waves, symbolizing these expectation-catalogs. If Schrödinger’s equation may be seen as “deterministic,” as it is sometimes, it is only in the sense that it strictly determines such expectation-catalogs, which are, however, catalogs of predictions that are not deterministic even in realist interpretations.

In his 1925 paper, introducing QM, Heisenberg began his derivation with an observation that reflects a radical departure from the classical ideal of continuous mathematical representation of individual physical processes, which would connect discrete quantum events. He says: “in quantum theory it has not been possible to associate the electron with a point in space, *considered as a function of time*, by means of observable quantities. However, even in quantum theory it is possible to ascribe to an electron the emission of radiation” [the effect of which is observed in a measuring instrument] [107]. Technically, a measurement could associate an electron with a point in space, but not by linking this association to a function of time representing the continuous motion of this electron, in the way it is possible in classical mechanics. Matrix mechanics did not offer a treatment of stationary states, when and only when one could in principle speak of the position of an electron in an atom, although while there are stationary energy states, with the same energy-levels, an electron itself is never stationary. Only an instantly repeated measurement can give the same value of its position, which instant repetition is an idealization. Heisenberg described his next task as follows: “In order to characterize this radiation we first need the frequencies which appear as functions of two variables. In quantum theory these functions are in the form” [107]:

*v*(*n*, *n* − α) = 1/*h* {*W*(*n*) − *W* (*n* − *α*)}

and in classical theory in the form

*v*(*n*, *α*) = *αv*(*n*) = *α*/*h*(*dW*/*dn*)

This difference leads to a difference between classical and quantum theories as regards the combination relations for frequencies, which, in the quantum case, correspond to the Rydberg-Ritz combination rules, reflecting, to return to Heisenberg’s locution, “the discrepancy between the calculated orbital frequency of the electrons and the frequency of the emitted radiation.” However, “in order to complete the description of radiation [in correspondence, by the correspondence principle, with the classical Fourier representation of motion] it is necessary to have not only frequencies but also the amplitudes” [107]. On the one hand, then, the new, quantum-mechanical equations must formally contain amplitudes, as well as frequencies. On the other hand, these amplitudes could no longer serve their classical physical function (as part of a continuous representation of motion) and are instead related to discrete transitions between stationary states. In Heisenberg’s theory and in QM since then, these “amplitudes” are no longer amplitudes of physical motions, which makes the name “amplitude” a *symbolic* term. In commenting on linear superposition in quantum mechanics in his classic book, Dirac emphasized this difference: “*the superposition that occurs in quantum mechanics is of an essentially different nature from any occurring in the classical theory*” [108]. In RWR-type interpretations, this superposition is not even physical: it is only mathematical. In classical physics the mathematics of (wave) superpositions represents physical processes; in QM, at least in the nonrealist view, it does not. Amplitudes are instead linked to the probabilities of transitions between stationary states: they are what became known as probability amplitudes. The corresponding probabilities are derived by a form of Born’s rule for this limited case (technically, one needs to use the probability density functions). The standard rule for adding the probabilities of alternative outcomes is changed to adding the corresponding amplitudes and deriving the final probability by squaring the modulus of the sum.

The mathematical structure thus emerging is in effect that of vectors and (in general, noncommuting) Hermitian operators in Hilbert spaces over **C**, which are infinite-dimensional, given that one deals with continuous variables. Heisenberg explains the situation in these terms in [100]. In his original paper, which reflect his thinking more directly, he argues as follows [107]:

The amplitudes may be treated as complex vectors, each determined by six independent components, and they determine both the polarization and the phase. As the amplitudes are also functions of the two variables *n* and *α*, the corresponding part of the radiation is given by the following expressions:

Quantum-theoretical:

Re{*A*(*n*, *n* − *α*)*e*^*i*ω(*n*, *n* − *α*)*t*^}

Classical

Re{*A_α_*(*n*)*e*^*iω*(*n*)*αt*^}

The problem—a difficult and, “at first sight,” even insurmountable problem—is that “the phase contained in *A* would seem to be devoid of physical significance in quantum theory, since in this theory frequencies are in general not commensurable with their harmonics” [109]. As noted, this incommensurability, which is in an irreconcilable conflict with classical electrodynamics, was one of the most radical features of Bohr’s 1913 atomic theory, on which Heisenberg builds here. His strategy is still based on the shift from calculating the probability of finding a moving electron in a given state to calculating the probability of an electron’s transition from one state to another, without describing the physical mechanism responsible for this transition. Heisenberg’s theory is more in harmony with this approach because there are no longer orbits, where the classical approach would still apply. 

Heisenberg says next: “However, we shall see presently that also in quantum theory the phase has a definitive significance which is *analogous* to its significance in classical theory” [109]. “Analogous” could only mean here that, rather than being analogous physically, the way the phase enters mathematically is analogous to the way the classical phase enters mathematically in classical theory, in accordance with the *mathematical* form of the correspondence principle, insofar as quantum-mechanical equations are formally the same as those of classical physics. Heisenberg only considered a toy model of an aharmonic quantum oscillator, and thus needed only a Newtonian equation for it, rather than the Hamiltonian equations required for a full-fledged theory, developed by Born and Jordan [8,110]. As Heisenberg explains, if one considers “a given quantity *x*(*t*) [a coordinate as a function of time] in classical theory, this can be regarded as represented by a set of quantities of the form” [109]:

*A_α_*(*n*)*e^iω^*^(*n*)*αt*^,

which, depending on whether the motion is periodic or not, can be combined into a sum or integral which represents *x*(*t*) [60]:

$$x(n,t)=\sum_{\alpha =-\infty }^{\alpha =+\infty }A_{\alpha }(n)e^{i\omega (n)\alpha t}$$

or:

$$x(n,t)=\int_{-\infty }^{+\infty }A_{\alpha }(n)e^{i\omega (n)\alpha t}d\alpha$$

Heisenberg next makes his most decisive and most extraordinary move. He notes that “a similar combination of the corresponding quantum-theoretical quantities seems to be impossible in a unique manner and therefore not meaningful, in view of the equal weight of the variables *n* and *n* − *α*. However, he says, “one might readily regard the ensemble of quantities *A*(*n*, *n* − *α*)e*^iω^*^(*n*, *n* − *α*)*t*^ [an infinite square matrix] as a representation of the quantity *x*(*t*)” [109]. The arrangement of the data into these ensembles, in effect square tables, was a remarkable way to handle the transitions between stationary states, although in retrospect it is also a natural way to do so, but only in retrospect. However, it does not by itself establish an *algebra* of these arrangements, for which one needs to find the rigorous rules for adding and multiplying these elements. Otherwise Heisenberg cannot use these variables in the equations of his new mechanics. To produce a quantum-theoretical version of the classical equation of motion considered, which would apply (no longer as an equation of motion) to these variables, Heisenberg needs to be able to construct the powers of such quantities, beginning with *x*(*t*)^2^, which is actually all that he needs for his equation. The answer in classical theory is obvious and, for the reasons just explained, obviously unworkable in quantum theory, where, Heisenberg proposes, “it seems that the simplest and most natural assumption would be to replace classical [Fourier] equations… by” [111]:

$$B(n,n-\beta )e^{i\omega (n,n-\beta )t}=\sum_{\alpha =-\infty }^{\alpha =+\infty }A(n,n-\alpha )A(n-\alpha ,n-\beta )e^{i\omega (n,n-\beta )t}$$

or:

$$=\int_{-\infty }^{+\infty }A(n,n-\alpha )A(n-\alpha ,n-\beta )e^{i\omega (n,n-\beta )t}d\alpha$$

This is the main mathematical postulate, the (matrix) multiplication postulate, of Heisenberg’s theory, “an almost necessary consequence of the frequency combination rules” [111].

Although it is commutative in the case of *x*^2^, this multiplication is in general noncommutative, expressly for position and momentum variables, and Heisenberg, without quite realizing, used this noncommutativity in solving his equation, as Dirac was the first to notice. Heisenberg spoke of his new algebra of matrices as the “new kinematics.” This was not the best choice of term because his new variables no longer described or were even related to motion as the term kinematic would suggest, one of many, historically understandable, but potentially confusing terms. Planck’s constant, *h*, which is a dimensional, dynamic entity, has played no role thus far. Technically, in Einstein’s view, the theory wasn’t even a mechanics: it did not offer a representation of individual quantum processes, but only predicted, probabilistically or statistically, what is observed in measuring instruments. To make these predictions, one will need Planck’s constant, *h*. 

That in general his new variables did not commute, *PQ* − *QP* ≠ 0, was, again, an especially novel feature of Heisenberg’s theory, confirming its essentially algebraic nature. This feature, which was, again, an automatic consequence of his choice of variables, proved to be momentous physically. Most famously, it came to represent the uncertainty relations constraining certain simultaneous measurements, such as those of the momentum (*P*) and the coordinate (*Q*), associated with a given quantum object in the mathematical formalism of quantum mechanics and (correlatively) the complementary character of such measurements. Given, however, the nature of the situation to which Heisenberg’s new mechanics responded, the noncommutative character of quantum variables should not be surprising. Schwinger instructively commented on the subject in his unpublished lecture, cited at length in [112]. He notes the most commonly stated physical feature corresponding to this character, namely, that if one measures two physical properties in one order, and then in the other, the outcome would in general be different. But he goes further in explaining why this is the case and its implications for the formalism [112]:

If we once recognize that the act of measurement introduces in the [microscopic] object of measurement changes which are not arbitrarily small, and which cannot be precisely controlled… then every time we make a measurement, we introduced a new physical situation and we can no longer be sure that the new physical situation corresponds to the same physical properties which we had obtained by an earlier measurement. In other words, if you measure two physical properties in one order, and then the other, which classically would absolutely make no difference, these in the microscopic realm are simply two different experiments…

So, therefore, the mathematical scheme can certainly not be the assignment, the association, or the representation of physical properties by numbers because numbers do not have this property of depending upon the order in which the measurements are carried out. … We must instead look for a new mathematical scheme in which the order of performance of physical operations is represented by an order of performance of mathematical operations.

This is not how Heisenberg discovered QM, in particular given that the noncommutativity of some among the operators representing quantum observables was not his starting point but a consequence of the multiplication rule for his matrices. The type of thinking described by Schwinger is more in accord with quantum-informational approaches to deriving quantum theory, primarily QTFD, from the (formalized) structure of quantum measurements, or as Schwinger revealingly put it the “measurement *algebra*.” [113,114]). The difficulty here is that any such scheme requires a mathematics that does not appear to be naturally connected to this measurement algebra, for one thing, because of the use of complex, rather than real, variables, and rules, such as Born’s rule, by means of which this scheme would be related to the probabilities or statistics of quantum predictions, which are real numbers. There is no homomorphic (let alone isomorphic) mapping from measurement algebra to the algebra of QM, “the new mathematical scheme.” One needs additional pieces of structure to arrive at this scheme. In Heisenberg, these additional pieces we partly borrowed from classical physics, formally defining his equations, and partly invented by Heisenberg in finding the variables needed. 

As explained earlier and as Schwinger stresses in his lecture, no identical assignment of the single quantity is ever possible, or in any event ever guaranteed, in two “identically” prepared experiments in the way it can be in classical physics [112]. This is because quantum experiments cannot be controlled so as to identically prepare quantum objects but only so as to identically prepare the measuring instruments involved, because this behavior can be considered classical. The quantum strata of measuring instruments, through which they interact with quantum objects, do not affect these preparations but only the outcomes of measurements. This interaction is uncontrollable. This fact is central to Bohr’s argument, which invokes this “finite and incontrollable interaction” at key junctures of his reply to EPR’s paper [34]. Hence, as noted earlier, the outcomes of repeated identically prepared experiments, including those involving sequences of measurements, cannot be controlled even ideally (as in classical physics), and these outcomes will, in general, be different. This circumstance makes statistical considerations unavoidable, as reflected, among other things, in the statistical character of the uncertainty relations, inherent in Heisenberg’s formula, Δ*q*Δ*p* ≅ *h*. The noncommutative nature of the corresponding variables responds to this character, along with the uncertainty relations themselves.

The quantum-mechanical situation that emerged with Heisenberg’s discovery of quantum mechanics and then Bohr’s interpretation of it was (sometime in the late 1930s) recast by Bohr in terms of his concept of “phenomenon,” defined by what is observed in measuring instruments under the impact of quantum objects, in contradistinction to quantum objects themselves, which could not be observed or represented, or in the present view, even conceived of. According to Bohr [115]:

I advocated the application of the word phenomenon exclusively to refer to the *observations* obtained under specified circumstances, including an account of the whole experimental arrangement. In such terminology, the observational problem is free of any special intricacy since, in actual experiments, all observations are expressed by unambiguous statements referring, for instance, to the registration of the point at which an electron arrives at a photographic plate. Moreover, speaking in such a way is just suited to emphasize that the appropriate physical interpretation of the symbolic quantum-mechanical formalism amounts only to predictions, of determinate or statistical character, pertaining to individual phenomena appearing under conditions defined by classical physical concepts [describing the observable parts of measuring instruments].

Phenomena are discrete in relation to each other, and, in Bohr’s scheme, one cannot assume that there are continuous processes that connect them, especially classically causally, even in dealing with elemental individual processes and events. Part of Bohr’s concept of phenomenon and the main reason for its introduction was that this concept “*in principle* exclude[s]” any representation or analysis, even if not a possible conception, of quantum objects and their behavior, at least, by means of QM [35]. The concept is, thus, correlative to the RWR-type view, reached by Bohr at this stage of his thinking, at least the weak RWR view. Physical quantities obtained in quantum measurements and defining the physical behavior of certain (classically described) parts of measuring instruments are *effects* of the interactions between quantum objects and these instruments, and do not pertain to quantum objects themselves. It is often forgotten by those who comment of Bohr’s insistence on the role of classical concepts in quantum theory that Bohr clearly realized and relied on the fact that the measuring instruments used in quantum experiments also have quantum parts through which they interact with quantum objects. Otherwise quantum measurements and the effects defining phenomena would not be possible. These effects are manifested by classical states of these parts of measuring instruments, to which these quantum interactions are “irreversibly amplified” [41]. The language of effects (in the absence of classical causes), found throughout Bohr’s writings on quantum mechanics, becomes especially prominent in his later articles, presenting his ultimate interpretation (e.g., [116]).

These effects are no longer assumed to correspond to any properties of quantum objects, even to single such properties, rather than only certain joint properties, in accordance with the uncertainty relations. An attribution of *even of a single property*, such as that of “position,” “moment in time,” “momentum,” or “energy,” or even invariant properties, such as the rest mass and charge of a particle, which are defined by the fact that they are the same in all measurements, to any quantum object is *never possible—before, during, or after measurement*. One could only rigorously specify measurable quantities physically pertaining to measuring instruments. Even when we do not want to know the momentum or energy of a given quantum object and thus need not worry about the uncertainty relations, neither the exact *position* of this object itself nor the actual time at which this “position” is established is ever available and, hence, in any way verifiable. Any possible information concerning quantum objects as independent entities is lost in “the finite [quantum] and uncontrollable interaction” between them and measuring instruments [34]. However, this interaction can leave a mark in measuring instruments, a mark, a bit of information, that can be treated as part of a permanent, objective record, which can be discussed, communicated, and so forth. The uncertainty relations, too, now apply to the corresponding (classical) variables of suitably prepared measuring instruments, impacted by quantum objects. We can either prepare our instruments so as to measure or predict a change of momentum of certain parts of those instruments or so as to locate the spot that registers an impact by a quantum object, but never do both in the same experiment. The uncertainty relations are correlative to the complementary nature of these arrangements.

Wheeler spoke of “law without law” in quantum theory [117]. One might see this concept, via the combination of the QPA and the RWR principles, as the algebraic probabilistic law of QM, without any law that would be assumed to govern the independent behavior of quantum objects. It is not surprising either that Wheeler eventually linked this “law without law” to quantum information theory, which he helped to usher, along with Feynman, his one-time student. Quantum objects, in their interactions with measuring instruments, create specifically organized collections of information (composed of classical bits) and make possible certain calculations, by using mathematical models, but we cannot know and possibly cannot conceive how quantum processes do this. The ultimate (quantum) constitution of matter is, according to Wheeler, “it from bit,” “it” inferred from “bit” [118]. In the present view, this “it,” while real, is beyond thought, and as such, cannot ultimately be called “it,” any more than anything else. Wheeler’s visionary manifesto was inspired by Bohr, whom Wheeler invoked on the same page that announced “it from bit:” “The overarching principle of 20th-century physics, the quantum—and the principle of complementarity that is the central idea of the quantum—leaves us no escape, Niels Bohr tells us, from ‘a radical revision of our attitude [towards the problem of] physical reality’” [118] (I correct Wheeler’s slight misquotation of Bohr).

Bohr’s argument for the necessity of this revision originated in Heisenberg’s algebraic method, of which Einstein, by contrast, remained ever skeptical, not the least because the concept of reality that was the product of this revision remained unpalatable to Einstein, or in his words, “while logically possible without contradiction, it [was] so contrary to [his] scientific instinct that [he] could not forego a search for a more complete conception” [119]. From Bohr’s perspective, QM is only incomplete when compared with the kind of realist knowledge possible in classical physics or relativity, which may be called the Einstein completeness. Otherwise, it is as complete as possible, as things stand now, which may be called the Bohr completeness. The question is whether nature would allow us to do better. While Einstein thought that it should, Bohr thought that it *might not*, which is not the same as it never will. As we haven’t heard nature’s last word on this matter, that is to say, nature’s next word (the only last word that nature gives us), the debate concerning this question continues with undiminished intensity.

## 5. From the Algebra of Circuits to the Algebra of Categories in Quantum Information Theory

Although Heisenberg’s creativity and inventiveness were remarkable and although it would be difficult to challenge him on the outcome, his derivation of QM may not have been as rigorous as one could ideally wish. While borrowing the *form* of equations from classical mechanics by the mathematical correspondence principle was a logical deduction concerning part of the mathematical structure of QM, Heisenberg virtually “guessed” the variables he needed. The mathematical expression of the principles in question was only partially worked out and sometimes more intuited than properly developed, which was in part remedied in the later work of Born, Jordan, and Heisenberg himself, but only in part. Even the derivation offered, following this more rigorous treatment, by Heisenberg in his Chicago lectures [100] might still be seen as falling short of a rigorous derivation from first principles, because it relied on intuitive moves of the type found in Heisenberg’s original derivation, especially as concerns his matrix variables, still essentially a guess and, arguably, the main difficulty for any rigorous (re)construction, especially for continuous variables. One could accordingly envision a more rigorous derivation. Most of the recent work in this direction has been in quantum information theory in dealing with discrete variables and finite-dimensional Hilbert spaces (QTFD). Some of these efforts, however, have affinities with that of Heisenberg, which, as I argue, exhibits a spirit of quantum-informational thinking. I shall now comment on two such cases, by D’Ariano and coworkers and by Hardy.

D’Ariano, Chiribella, and Perinotti’s (DACP’s) program, developed over the last decade and presented comprehensively in their book [82], belongs to a particular trend in quantum information theory, and as most of the work there, it deals with discrete variables and the corresponding, finite-dimensional, Hilbert spaces (e.g., [120,121,122]). This is in part because, as DACP note (a view shared by others in this field), “the study of finite-dimensional systems allows one to decouple the conceptual difficulties in our understanding of quantum theory from the technical difficulties of infinite-dimensional systems” [123]. A rigorous (or at least more rigorous than that of founding figures) derivation of QM, let alone QFT, from fundamental principles remains an open and difficult task, to which I return below. 

DACP’s project is motivated by “a need for a deeper understanding of quantum theory [QTFD] in terms of fundamental principles,” and by the aim of deriving QTFD from such principles, which, the authors contend, has never been quite achieved by their predecessors. As indicated earlier, the fluctuations of terms such as principles, axioms, postulates, commonly used in these reconstructive projects may be confusing and obscure this common aim. DACP use the term “axioms” as well. On the other hand, while one can surmise their understanding of the term “principle” from their use of it, they do not define the concept of “principle” either. As earlier, I adopt the concept of principle defined above, via Einstein. This concept is, I would argue, in accord with DACP’s use of principles, and their derivation of QTFD, or that of Hardy, may be seen as that of a principle theory in Einstein’s sense.

I put aside the question to what degree this (or Hardy’s) derivation amounts to a *fully* rigorous derivation, which, or in the first place, the question of what could be considered as a fully rigorous derivation, would require a separate analysis. One might even question the necessity of a “*fully* rigorous definition.” After all, Heisenberg, at least as his scheme was developed by Born and Jordan, and then differently by Dirac, did establish a correct theory, and then Dirac similarly invented QED, and various parts of QFT were created similarly. Heisenberg in effect posed this question in commenting on his own derivation of QM: “It should be distinctly understood, however, this [deduction of the fundamental equation of quantum mechanics] cannot be a deduction in the mathematical sense of the word, since the equations to be obtained form themselves the *postulates* of the theory. Although made highly plausible by the following considerations [of the type that led him to his discovery of QM], their ultimate justification lies in the agreement of their predictions with the experiment” [124]. While a derivation of QM, or QTFD, might be made more rigorous than that offered by Heisenberg even there, it is doubtful that any such derivation, *from (physical) first principles*, could ever be as rigorous as “a deduction in the mathematical sense of the word.” As Hardy suggested, even by his title, in [120], it may be more a matter what are “reasonable” initial axioms or postulates, although such a “reasonableness” is not a simple or unconditional matter either. It goes without saying that these qualifications in no way diminish the significance of DACP’s or Hardy’s work, or that of others pursuing this line research. Besides, as I shall explain, more than merely re-deriving QTFD, or QM and QFT is at stake in these programs.

The main new feature of DACP’s approach is adding to the view of QTFD as an extension of probability theory (a view found in the works of their predecessors and applicable to QM as well) “the crucial ingredient of *connectivity* among events” by using the operational framework of “circuits” and giving it an algebra [125]. The framework of circuits has been similarly used by others, specifically by Hardy. This addition allows DACP “to derive key results of quantum information theory and general features of quantum theory [QTFD]” without first assuming Hilbert spaces. Unlike von Neumann, to whom DACP refer for a contrast, Heisenberg, as we have seen, did not begin with a formalism either, although he used the equation of classical mechanics by the correspondence principle. He *arrived* at his formalism from fundamental principles, even if, again, not fully rigorously *derived* it from these principles. 

Among the principles adopted by DACP, the purification principle plays a unique role as an essentially quantum principle, because conforming to it distinguishes QTFD from classical probabilistic information theories. According to them: “The purification of mixed states is specifically quantum” [126] (it may be a question whether it *uniquely* quantum, on which I shall comment presently). It is the single principle necessary to do so, which may not be surprising given the history of quantum theory and attempts at its axiomatic derivations. Hardy’s pioneering derivation also needed only one axiom, the continuity axiom, to do so [120]. On the other hand, that Hardy’s continuity axiom is different from DACP’s purification postulate suggests that there may not be a single system of postulates or principles from which QTFD could be derived, even though all such systems should capture something that pertains uniquely to quantum phenomena, and thus to quantum objects, even if one assumes that they are beyond representation or conception. Their effects are representable and enable one to distinguish classical and quantum phenomena, and infer from them the existence of quantum objects. 

Whether one can do so definitively remains a question, noted from the outset in connection with Planck’s constant, *h*. While this question and literature concerning it are beyond my scope, I would like to comment on Spekkens’s recent work, which is especially relevant here because it proceeds along the lines of quantum information theory. Spekkens introduced several toy models or theories, “epirestricted theories” (so-called because of epistemic restrictions on the classical theory, assumed as a starting point), that reproduce many quantum phenomena and features of QM or QTFD, such as the presence of *h*, the uncertainty relations, noncommuting operator observables, entanglement, or the purification of mixed states [127,128,129]. Many but not all! These models expressly fail to reproduce some among the crucial features of quantum theory, specifically some of those dealing with correlations and entanglement, such as violations of Bell inequalities and the existence of the Kochen-Specker theorem, which is, however, predictable given the nature of Spekkens’s models, as Spekkens explained [130]. This is crucial because these theorems reflect the essential features of quantum phenomena (independently of any theory, which, however, must, if correct, satisfy them), and are also crucial to the question of realism and locality, all of which is noted by Spekkens [130]. Accordingly, whether actual quantum phenomena found in nature can be captured by models of this type remains an open question. According to Spekkens himself, this is unlikely [131]:

The investigation of epirestricted theories, therefore, need not—and indeed *should not*-be considered as the first step in a research program that seeks to find a ψ-epistemic ontological model [a realist model that assumes quantum states in the formalism to be epistemic, states of knowledge] of the full quantum theory. Even though such a model could always circumvent any no-go theorems by violating their assumptions, it would be just as unsatisfying as a ψ-ontic model [a realist model that assumes quantum states to be ontic, states of reality] insofar as it would need to be explicitly nonlocal and contextual. Rather, the investigation of epistricted theories is best considered as a first step in a larger research program wherein the framework of ontological models—in particular the use of classical probability theory for describing an agent’s incomplete knowledge—is ultimately rejected, but where one holds fast to the notion that a quantum state is epistemic.

Although common in quantum information theory, viewing quantum states (state vectors in the formalism) as states of knowledge could be misleading, unless one strictly refers to this knowledge as probabilistic or statistical. (Even then quantum states are only part of the corresponding predictive machinery, as one needs Born’s or related rule, such as Lüders’ postulate, to have these probabilities or statistics.) As noted earlier, from the present, RWR-type perspective, in which quantum states in this mathematical sense never refer to any actual, determined knowledge (which is only obtained in measurements), our knowledge, actual (obtained in measurement) or probabilistic is incomplete only when compared with the kind of realist knowledge possible in classical physics or relativity—the Einstein completeness. Otherwise, it is as complete as possible, as things stand now—the Bohr completeness.

I am not sure whether, his skepticism concerning ontological models of quantum theory notwithstanding, Spekkens would be willing to go that far, especially to the strong RWR view. It appears, however, at least to the present reader, that his epirestricted theories and his argument suggest that the difference between the classical and the quantum may ultimate be irreducible, even though finding and rigorously grounding reconstructive programs remains difficult. According to Spekkens, “it may be that there are particularly elegant axiomatic schemes that are not currently in our reach and the road to program involves temporarily setting one’s sight a bit lower,” moving to partial reconstructive models of the kind he proposes [132]. That may be, especially in the case of continuous variables, to which Spekkens’s epirestricted theories apply. DACP’s or Hardy’s approach may suggest otherwise, even while dealing only with QTFD. There is also a question whether such elegance should be the main criterion here, especially given that one’s goals may ultimately be beyond QTFD or QM? While Dirac would have thought so, neither Heisenberg (in his derivation) nor Bohr was much worried about elegance. It is difficult to be certain which trajectories will lead us there, especially if this “there” is beyond QTFD, as it ultimately must. There may not be one such trajectory, just as in the discovery of QM, there were two—Heisenberg’s and Schrödinger’s. I leave this complex set of subjects on the following note, concerning the role of *h*.

It is true that there are classical phenomena, in considering which *h* must be taken into account, even trivially true, although Spekkens’s model considered in [129] is nontrivial because it involves other quantum-like features. On the other hand, most classical phenomena do not involve *h* at all, while *all* quantum phenomena known thus far require taking *h* into account, just as all (special) relativistic account require taking *c* into account. This does, I think, tell us something about the nature of the quantum or our technological interactions with that which we call the quantum, even if does not tell us the whole story, assuming that such a story can ever be told. If one adopts an RWR-type view, there is no story to be told about how quantum phenomena come about, and *h*, too, may only appear in our interaction with nature and not be a property of nature itself. That, however, does not mean that more cannot be said, perhaps even definitively, about what distinguishes quantum from classical phenomena. I now return to DACP’s derivation of QTFD and the purification principle, which they see as “specifically quantum”.

In nontechnical terms, the purification principle states that “every random preparation of a system can be achieved by a pure preparation of the system with an environment, in a way that is essentially unique” [133]. The principle originates in Schrödinger’s insight in his response, in several papers, to the EPR paper and his concept of entanglement [72]. According to DACP [134]:

The purification principle stipulates that, whenever you are ignorant about the state of a system A, you can always claim that your ignorance comes from the fact that A is part of a large [composite] system AB, of which you have full knowledge. When you do this, the pure state that you have to assign to the composite system AB is determined by the state of A in an essentially unique way.

The purification of mixed states is a peculiar feature—surely, not one that we experience in our everyday life. How can you claim that you know A *and* B if you don’t have A alone? This counterintuitive feature has been noted in the early days of quantum theory, when Erwin Schrödinger famously wrote: “Another way of expressing the peculiar situation is: *the best possible knowledge of a whole does not necessarily include the best possible knowledge of all its parts.*” And, in the same paper: “I would not call that *one* but rather *the* characteristic trait of quantum mechanics, the one that enforced its entire departure from classical lines of thought …”

[135]

The purification of mixed states is specifically quantum. But why should we assume it as a fundamental principle of Nature? At first, it looks like a weird feature—and it must look so, because quantum theory itself is weird and if you squeeze it inside a principle, it is likely that the principle looks weird too. However, on second thought one realizes that purification is a fundamental requirement: essentially, it is the link between physics and information theory. Information theory would not make sense without the notions of probability and mixed state, for the whole point about information is that there are things that we do not know in advance. But in the world of classical physics of Newton and Laplace, every event is determined and there is no space for information at the fundamental level. In principle, it does not make sense to toss a coin or to play a game of chance, for the outcome is already determined and, with sufficient technology and computational power, can always be predicted. In contrast, purification tells us that “ignorance is physical.” Every mixed state can be generated in a single shot by a reliable procedure, which consists of putting two systems in a pure state and discarding one of them. As a result of this procedure, the remaining system will be a physical token of our ignorance. This discussion suggests that, only if purification holds, information can aspire to a fundamental role in physics.

Technically, the purification of mixed states is the fundamental principle arising in *our interactions with nature* by means of our experimental technology, rather than of nature itself, except insofar as we and our technologies are also nature. But then, in the present view, there is no other principles of nature than those defined by us in our interactions with it. DACP’s formulation of the purification principle and their derivation of QTFD allows for an RWR-type interpretation, and I shall interpret it in this way, without claiming that this necessarily corresponds to DACP’s own view.

Thinking in terms of “circuits” is close to Bohr’s thinking concerning the role of measuring instruments in the constitution of quantum phenomena, as distinguished from quantum objects, which give rise to quantum phenomena by interacting with measuring instruments but which are never observable. Circuits and their arrangements, too, embody those of measuring instruments capable of detecting quantum events, and thus enabling the probabilistic predictions of future events. Their arrangements and operations, defining their “measurement algebra,” are enabled by rules that should ideally be derived from certain sufficiently natural assumptions. They are described classically, and thus embody the structure of quantum information as a particular form of organization of classical information, which can be used, as by DACP (or Hardy), to *derive* the mathematical formalism of QTFD.

While indispensable for the authors’ derivation of QTFD, the purification principle is not sufficient to do so. They need five additional postulates (termed “axioms”): *causality* (essentially locality), *local discriminability*, *perfect distinguishability*, *ideal compression*, and *atomicity of composition* [133]. These postulates define a large class of classical probabilistic informational theories, while the purification postulate, giving rise to the purification principle, distinguishes QTFD. The appearance of these additional postulates or principles is not surprising. Heisenberg’s grounding principles, the quantum discreteness (QD) principle and the quantum probability or statistics (QP/QS) principle, were not sufficient for him to derive QM either. To do so, he needed the correspondence principle, which gave him half of the mathematical architecture of quantum theory. The other half was supplied by his matrix variables.

There are instructive parallels between DACP’s and Heisenberg’s approaches. Both the QD and QP/QS principles are present in both cases. As they say in their earlier article: “The operational-probabilistic framework combines the operational language of circuits with the toolbox of probability theory: on the one hand experiments are described by circuits resulting from the connection of physical devices, on the other hand each device in the circuit can have classical outcomes and the theory provides the probability distribution of outcomes when the devices are connected to form closed circuits (that is, circuits that start with a preparation and end with a measurement)” [136]. This is similar to Heisenberg’s thinking in his paper introducing QM, as the classical outcomes are discrete in both cases as well. The concept of “circuit” is not found in Heisenberg and is, again, closer to Bohr’s view of the role of measuring instruments and his concept of phenomenon, defined by this role. As I explained, however, the idea that in quantum theory we only deal with transition probabilities between the outcomes of the interactions between quantum objects and measuring instruments was introduced by Heisenberg, as part of his approach to QM, and was then adopted by Bohr. Heisenberg discovered that Bohr’s frequencies rules are satisfied by, in general, non-commuting matrix variables with complex coefficients, from which one derives, by means of a Born-type rule, probabilities or statistics for transitions between stationary states, manifested in spectra observed in measuring devices. Thus, Heisenberg’s derivation depended on measuring instruments as devices with classically describable observable parts, which are akin to “operational circuits,” in his case dealing with continuous variables.

DACP aim to arrive at the mathematical structure of QTFD in a more first-principle-like way, for example, independently of classical physics, which, because of the correspondence principle, was central to Heisenberg (classical physics, to begin with, does not have discrete variables, so there is no correspondence principle). The rules governing the structure of operational devices, circuits, should, they argue, allow them to do so, because these rules are *empirical*. They are, however, *not completely empirical*, because circuits *are given* a mathematical structure, in effect algebra, by human agents, even though this algebra may be partially defined by the organization, required by experiments, of the experimental arrangements in which circuits appear. The mathematized structure or algebra of circuits become a necessary condition for establishing the mathematical structure or algebra of QTFD, but it is not sufficient to do so. As noted above in considering Schwinger’s argument, these two structures are not isomorphic or even homomorphic, and they do not appear to be in DACP’s derivation. This means that additional pieces of structure, provided by additional postulates or axioms, are required for one thing, to get to the (Hilbert-space or other) formalism over **C**. Then, one needs a Born-type rule for the probabilities of predictions. DACP’s derivation requires enormous technical work. It is next to impossible to do it justice here. I shall instead close the article by considering the algebra of circuits in considering Hardy’s work.

Hardy has at a different set of main assumptions necessary to derive QTFD than those of DACP, but the main strategy is the same: establishing the architecture, algebra, of circuits that, with additional axioms, would allow one to derive the mathematical formalism of QTFD. According to Hardy [137]:

Circuits have:

- A setting, s(H), given by specifying the setting on each operation.
- An outcome set, o(H), given by specifying the outcome set at each operation (equals o(A) × o(B) × o(C) × o(D) × o(E) in this case). We say the fragment “happened” if the outcome is in the outcome set.
- A wiring, w(E), given by specifying which input/output pairs are wired together.

With this algebraic definition in hand, I shall comment on some of Hardy’s fundamental assumptions discussed by him in a different paper. Hardy says [138]:

We will make two assumptions to set up the framework in this paper…

**Assumption** **1.** *The probability, Prob (A), for any circuit, A (this has no open inputs or outputs), is well conditioned. It is therefore determined by the operations and the wiring of the circuit alone and is independent of settings and outcomes elsewhere.*

This is a physical postulate, essentially that of locality, combined with probability or statistics, along the lines of the QP/QS principle. The task now becomes how to derive a QTFD that could correctly predict these probabilities. One needs another assumption:

**Assumption** **2.** ***Operations are fully decomposable…** We assume that any operation $A_{a1b2\ldots c3}^{d4e5\ldots f6}$ can be written, … in a symbolic notation, $A_{a1b2\ldots c3}^{d4e5\ldots f6}\;\equiv^{d4e5\ldots f6}A_{a1b2\ldots c3}X_{a1}^{a1}X_{b2}^{b2}\ldots X_{c3\;d4}^{c3}{\;X}_{\;e5}^{d4}X_{...f6}^{e5}X^{f6}.$ In words we will say that any operation is equivalent to a linear combination of operations each of which consists of an effect for each input and a preparation for each output… We allow the possibility that the entries in _d4e5…f6_A_a1b2…c3_ are negative (and this will, indeed, be the case in quantum theory). Hence, in general, this cannot be thought of as physical mixing… (emphasis added).*

I only need the italicized sentence for my conceptual point, insofar as it means that one can construct a suitable algebra. I cite the passage at a greater length to illustrate a manifested algebraic view of circuits and operations in Hardy’s scheme. Hardy then says [138]:

Assumption 2 introduces a subtly different attitude than the usual one concerning how we think about what an operation is. Usually we think of operations as effecting a transformation on systems as they pass through. Here we think of an operation as corresponding to a bunch of separate effects and preparations. We need not think of systems as things that preserve their identity as they pass through—we do not use the same labels for wires coming out as going in. This is certainly a more natural attitude when there can be different numbers of input and output systems and when they can be of different types. Both classical and quantum transformations satisfy this assumption. In spite of the different attitude just mentioned, we can implement arbitrary transformations, such as unitary transformations in quantum theory, by taking an appropriate sum over such effect and preparation operations.

This rethinking of the concept of operation is important, especially if one adopts an RWR-type view. An “operation” is now defined in terms of observable “effects” of the interactions between quantum objects and measuring instruments, and not in terms of what happens, even in the course of these interactions (let alone apart from them), to the quantum objects or systems, *considered as independent systems*. It is useful that we can treat classical systems in this way as well. In the classical case, however, we can, equivalently, use a more conventional concept of operation mentioned here, which is not the case in quantum theory. After a technical discussion of “duotensors,” which I put aside, Hardy suggests a principle [139]:

*Physics to mathematics correspondence principle*. For any physical theory, there [exist] a small number of simple hybrid statements that enable us to translate from the physical description to the corresponding mathematical calculation such that the mathematical calculation (in appropriate notation) looks the same as the physical description (in appropriate notation).

Such a principle might be useful in obtaining new physical theories (such as a theory of quantum gravity). Related ideas to this have been considered by category theorists. A category of physical processes can be defined corresponding to the physical description. A category corresponding to the mathematical calculation can also be given. The mapping from the first category to the second is given by a functor (this takes us from one category to another).

The language of correspondence should not mislead one into relating this principle to Bohr’s correspondence principle, even in Heisenberg’s *mathematical* form. Bohr’s correspondence principle deals with the correspondence between different physical theories (such as classical mechanics and quantum mechanics), insofar as their predictions would coincide in the regions when both theories could be used. By contrast, apart from the fact that, as explained, there is no correspondence principle in QFDT, Hardy’s “hybrid” construction implies that category of physical processes could be functorially “translated” into a proper formalism of QTFD, which can then, through mathematical calculations enabled by this formalism, be related to what is observed. Hardy’s principle may be better called “physics to mathematics functoriality principle.” Hardy’s suggestion, inviting but somewhat speculative and not really worked out, to begin with, would require a separate discussion, which cannot be undertaken here. It is worthwhile, however, to offer a few brief comments, via the role of category theory in the algebraization of physics, without definitively claiming that these comments are fully in accord with Hardy’s view of the situation.

Category theory originated in algebraic topology and then was extensively used, especially thanks to Grothendieck’s work, in algebraic geometry, in order to study certain algebraic invariants, such as cohomology or homotopy groups, associated with topological spaces [140]. It was then extended, via topos theory, introduced by Grothendieck as well, to mathematical logic, a subject I shall put aside here [141]. Roughly, category theory considers multiplicities (categories) of mathematical objects conforming to a given concept, such as the category of topological spaces or geometrical spaces (say, Riemannian manifolds), and the morphisms, also called arrows (X→Y), which are the mappings between these objects that preserve this structure. Studying morphisms allows one to learn about the individual objects involved more than we would by considering them individually. Thus, in geometry one does not have to start with a Euclidean space. Instead the latter is just one specifiable object of a large categorical multiplicity, such as the category of Riemannian manifolds, an object marked by a particularly simple way we can measure the distance between points. Categories themselves may be viewed as such objects, and in this case one speaks of “functors” rather than “morphisms.”

Now, one can more easily think of properly defining mathematically, the second category in Hardy’s suggestion, say, as that of Hilbert spaces [142] or some more directly categorical equivalent algebra by means of which the mathematical calculations in question would be performed. On the other hand, the first category, that is, the structure of its objects and the morphisms between them, and, thus, the nature of the functor in question between these two categories is a more complex matter. Hardy’s formulations above “for any physical theory, there [exist] a small number of simple hybrid statements that enable us to translate from the physical description to the corresponding mathematical calculation such that the mathematical calculation (in appropriate notation) *looks the same* as the physical description (in appropriate notation)” (emphasis added) might require qualification as concerns the meaning of the expression “looks the same” and the relationships between “calculation” and “description,” because this formulation allows for different interpretations. The same may be said about his characterization of the functor in question as “*virtually* direct” (emphasis added) and then the statement “a category of physical processes can be defined corresponding to the physical description.”

I shall sketch the reasons for these qualifications, beginning with a possible meaning of “*physical processes*” in “a category of physical processes can be defined corresponding to the physical description.” “Physical processes” may refer either to quantum processes, or to circuits, which appears more likely because “the physical description” in Hardy’s scheme (or that of DACP) is given only at the level of circuits. This view would also be suggested by the category theorists who work on using categories in quantum theory, specifically QTFD, such as Abramsky, Coecke, and others, to whom Hardy appears to refer here. Most of this work is primarily concerned with recasting the Hilbert-space language of the QTFD formalism in a categorical framework by replacing Hilbert spaces, belonging to the category of the finite-dimensional Hilbert spaces over **C** (e.g., [142]), with objects of monoidal categories and the morphism between Hilbert spaces with morphism between these objects, rather than with deriving the formalism from the first (physical) principles. There are some moves in this direction, say, by starting with a suitable simple monoidal category and then consider which additional pieces of structure one needs to arrive at QTFD (e.g., [143]). This work further testifies to the dominant role of algebra in quantum theory, even though it uses a lot of diagrammatic operations, somewhat akin to Feynman diagrams in QFT. As Coecke and Kissinger’s title, “picturing quantum processes,” indicates, they appear to hold a realist view in assuming that their diagrammatic picturalism represents the actual quantum behavior, which assumption, I argue here, has complexities, in part because of the role of complex quantities, complexities not addressed by Coecke and Kissinger [143]. In RWR-type views, quantum behavior is beyond representation or even conception, and thus pieces of this behavior (say, between measurements) cannot be assumed to form a category. Hardy’s position on this point is not entirely clear. If quantum processes themselves are given some representation, the corresponding category may need to incorporate this representation in one way or another, by combing it with the effects of quantum processes manifested in circuits and their arrangements.

If one adopts an RWR-type view (either weak or strong), one only deals with the physical description of circuits, similarly to dealing only with the description of measuring instruments, according to Bohr. Circuits and their organizations, their algebra, again, embody the arrangements of measuring instruments capable of detecting quantum events and enabling the probabilistic or statistical predictions of future events, in other words, a structure that may be mathematizable and as such *categorially* translatable into the mathematical formalism of QTFD (which enables the necessary mathematical calculations). As discussed earlier in considering both Schwinger’s argument and DACP’s derivation of QTFD, one need not and, in the RWR-type view, should not expect a representational correspondence between the structures or algebra of circuits and the mathematical structures or the algebra of the formalism of QTFD. For one thing, one needs, again, a formalism, Hilbert-space one, C*-algebra, categorical, or other, over **C**. As classical, the data manifested in circuits is over **R** (in fact, all measurements are rational numbers, while probabilities need not be). Then, one needs a Born-type rule to get to probabilities. It follows, then, as, again, considered earlier, that one needs additional pieces of structure to those defined by circuits.

One could contemplate two approaches. The one, closer to QTFD category theorists, is to use these additional pieces of structure (algebraic relations, axioms, etc.) to build a category of algebraic mathematical objects, which need not be Hilbert spaces, but would, again, have to be over **C**, and rules, which enable proper probabilistic or statistical predictions of quantum phenomena, again, observed in circuits [143]. Alternatively, closer to what Hardy appears to suggest and to Bohr’s way of thinking, one could attempt to establish a category of circuits, each defined by an algebraic structure of units and operations, and morphisms between them, and then, again, by using some reasonable axioms, a category of objects defining the formalism, such as Hilbert spaces or some monoidal categories, and morphism between them (along, again, with a Born-like rule for probabilities), and then connect these two categories by means of a well-defined functor. The advantage of the second approach is that it preserves the role of the algebraic structure of circuits, which are observable. The difficulty is, again, that there is no natural categorical structure for the algebra of circuits, morphisms, etc., all of which need to be defined, which, however, is also true in the first approach. To do so requires additional pieces of structure, and thus additional more or less reasonable principles or postulates, rather than intuitive guesses, assuming, again, that the latter could be entirely avoided. In the second approach (which I prefer), it is the question of a functorial relationship between categories, which, again, still needs to be established, rather than morphism between objects, such as the algebras of circuits and the algebras of the formalism of QTFD, or by implication, QM or QFT, a more complex project. There are morphisms in each category, and there functors between categories, and the view just considered is about the latter. From this perspective, Hardy’s “physics to mathematics correspondence principle” would, if rigorously established, be the “physics to mathematics *functoriality* principle”: it would be realized in terms of the functorial relationship between a category based on circuits (the objects and morphisms of which need, again, to be defined) and a category of the algebraic objects defining the formalism, such as that of Hilbert spaces over **C**, finite-dimensional ones in QTFD, or some other category of objects and morphisms. 

One of the most essential aspects of category theory, even its *raison d’être*, at least in its use in algebraic topology and algebraic geometry (mathematical logic is, again, a different matter), consists in establishing the relationships between multiplicities, “categories,” of objects of *different* types, for example, geometrical or topological objects and algebraic objects, or between algebraic objects of different types (such as Lie groups and Lie algebras), rather than, apart from trivial cases, directly mapping the objects of the first category on those of the second. Indeed, the concept of category was introduced in the field of algebraic topology and then extended to algebraic geometry to help study certain algebraic invariants, such as groups, associated with topological spaces. In contradistinction to geometry, defined, as a mathematical discipline, by the concept of measurement (geo-*metry*), topology, as a mathematical discipline, is defined by associating an algebraic structure or a set of structures, most especially groups, such as homotopy or cohomology groups (which came to play an important role in QFT) to a topological space. The structure of a topological space is defined by its continuities and discontinuities, and not by its geometry, even if it had a geometry, and not all topological spaces do. Insofar as one deforms a given figure continuously (i.e., insofar as one does not separate points previously connected and, conversely, does not connect points previously separated), the resulting figure or space is treated as mathematically equivalent. Thus, no matter how much you expand or continuously (without separating connected points or joining separated points) deform the two-dimensional surface of a sphere the resulting spaces are topologically equivalent (homeomorphic), while some of these objects are no longer spherical geometrically speaking. Such spaces are, however, topologically distinct from those of topologically deformed two-dimensional surfaces of tori because spheres and tori cannot be converted into each other without disjoining their connected points or joining the separated ones: the holes in tori make this impossible. By contrast, the spaces of spheres (or any spaces) of different dimensions are not homeomorphic. In algebraic topology, mathematical objects of each type are arranged in categories and are related to one another by morphisms, while categories are related by functors. The category of topological spaces (or a subcategory, such as that of Riemannian manifolds) and their morphisms becomes related to a category of algebraic objects, such as groups and their morphisms. The relationships between these two categories, topological and algebraic, allow one to extract an enormous amount of information concerning topological spaces and, conversely, groups (through the structure of topological spaces to which these groups are associated). A good categorical approach to, and a possible derivation of, quantum theory would similarly establish the functorial relationships between two, now algebraic, categories, one based in circuits and the other defining the formalism.

An important and difficult question, which need not depend on a categorical formalization, but may be helped by it, is that of the relationship between the structure of the circuits, defined by the corresponding experimental arrangements, and the infinite-dimensional mathematical architecture of QM, or the same relationship in QFT. Consider the double-slit experiment, say, in the interference pattern setup. It is a circuit, which embodies preparations, measurements, and predictions, all manifested in the emergence of the interference pattern. I would not presume to be able to mathematize it. But it is a circuit nevertheless, a complex one, albeit child’s play in comparison to the circuitry found in high-energy quantum physics, such as that of the Large Hadron Collider (LHC), which led to the detection of the Higgs boson.

Such questions will need be addressed if one is to extend the programs to derivations of QTFD to QM or to QED and QFT, or beyond. Indeed, it is hardly sufficient to merely derive already established theories. The ultimate value of these programs lies in what they can do for the future of fundamental physics. Thus, Hardy aims to rethink general relativity in operational terms, analogous to those of QTFD, and then to reach, in principle, quantum gravity, bypassing QFT or even QM, which would then be merely special cases of the ultimate theory [16,83]. D’Ariano and co-workers, by contrast, appear first to move from QTFD to QFT. In their more recent work, they aim to develop a new approach to QFT, which, as based on the concept of quantum cellular automata, is different from the operational framework discussed thus far, but shares with it certain key informational features and, most especially, the aim of developing QFT from fundamental (first) principles. D’Ariano and Perinotti’s derivation of Dirac’s equation is a step in this direction, for now in the absence of an external field, essential to the proper QED [144,145]. Unlike Dirac’s own or other previous derivations of the equation, their derivation only uses, along with other principles (homogeneity, isotropy, and unitarity), the principle of locality, rather than special relativity. The approach may, they hope, offer new possibilities for fundamental physics on Planck’s scale, suggesting a potential extension of quantum information theory as far as one can envision it now.

Whether the QPA principle (which is in part experimental, empirical) and the RWR principle (which is interpretive) will remain viable or will be defeated, fulfilling the hope of Einstein and those who followed him, is an open question. On the other hand, it appears likely, as no currently known attempts to move beyond QFT (such as string and M-brane theory, or loop quantum gravity) would indicated otherwise, that we will see the emergence of new algebraic and spatial-algebraic structures. We very much need them for quantum gravity, for example. Because algebraic topology and algebraic geometry are likely to play a role in quantum gravity, the age of algebra—the age of Fermat, Descartes, and Leibniz—is likely to continue for the foreseeable future in mathematics and physics alike.
